# Antimicrobial, Cytotoxic, and *α*-Glucosidase Inhibitory Activities of Ethanol Extract and Chemical Constituents Isolated from *Homotrigona apicalis* Propolis—In Vitro and Molecular Docking Studies

**DOI:** 10.3390/life13081682

**Published:** 2023-08-03

**Authors:** Diep Thi Lan Phuong, Nguyen Van Phuong, Nguyen Le Tuan, Nguyen Thanh Cong, Nguyen Thu Hang, Le Nguyen Thanh, Vu Thi Hue, Nguyen Quoc Vuong, Nguyen Thi Thu Ha, Milena Popova, Boryana Trusheva, Vassya Bankova

**Affiliations:** 1 Faculty of Natural Sciences, Quy Nhon University, Binh Dinh 55000, Vietnam; nguyenletuan@qnu.edu.vn; 2 Department of Pharmacognosy, Faculty of Pharmacognosy and Traditional Medicines, Hanoi University of Pharmacy, Hanoi 11000, Vietnam; phuongnv@hup.edu.vn (N.V.P.); thanhcong11789@gmail.com (N.T.C.); hangnt@hup.edu.vn (N.T.H.); 3 Department of Pharmacy, Dai Nam University, Hanoi 10000, Vietnam; 4 Graduate University of Science and Technology, Vietnam Academy of Science and Technology (VAST), Hanoi 10000, Vietnam; nguyenvh62@gmail.com (N.Q.V.); thuha.vast@gmail.com (N.T.T.H.); 5 Institute of Marine Biochemistry, Vietnam Academy of Science and Technology (VAST), Hanoi 10000, Vietnam; vuhue.hnue@gmail.com; 6 Institute of Chemistry, Vietnam Academy of Science and Technology (VAST), Hanoi 10000, Vietnam; 7 Institute of Organic Chemistry with Centre of Phytochemistry, Bulgarian Academy of Sciences, Acad. G. Bonchev Str., bl. 9, 1113 Sofia, Bulgaria; milena.popova@orgchm.bas.bg (M.P.); boryana.trusheva@orgchm.bas.bg (B.T.); vassya.bankova@orgchm.bas.bg (V.B.)

**Keywords:** *Homotrigona apicalis*, sesquiterpenes, triterpenes, xanthones, *α*-glucosidase, cytotoxicity

## Abstract

The chemical investigation of *Homotrigona apicalis* propolis collected in Binh Dinh province, Vietnam, led to the isolation of nine compounds, including four sesquiterpenes: spathulenol (**1**), 1*α*H,5*β*H-aromandendrane-4*β*,10*α*-diol (**2**), 1*β*,6*α*-dihydroxy-4(15)-eudesmene (**3**), and 1*β*H,5*β*H-aromandendrane-4*α*,10*β*-diol (**4**); three triterpenes: acetyl oleanolic acid (**5**), 3*α*-hydroxytirucalla-8,24-dien-21-oic acid (**6**), and ursolic acid (**7**); and two xanthones: cochinchinone A (**8**) and *α*-mangostin (**9**). Sesquiterpens **1**–**4** and triterpene **6** were isolated for the first time from stingless bee propolis. Plants in the *Cratoxylum* and *Aglaia* genus were suggested as resin sources of the propolis sample. In the antibacterial activity evaluation, the EtOH extract only showed moderate activity on *S. aureus*, while the isolated compounds **7**–**9** showed good antibacterial activity, with IC_50_ values of 0.56 to 17.33 µg/mL. The EtOH extract displayed selective cytotoxicity against the A-549 cancer cell line, with IC_50_ values of 22.82 ± 0.86 µg/mL, and the xanthones **8** and **9** exhibited good activity against the KB, HepG-2, and A-549 cancer cell lines, with IC_50_ values ranging from 7.55 ± 0.25 µg/mL to 29.27 ± 2.07 µg/mL. The cytotoxic effects of xanthones **8** and **9** were determined by the inhibition of the EGFR and HER2 pathways using a molecular docking study. Compounds **8** and **9** displayed strong binding affinity with EFGR and HER2, with values of −9.3 to −9.9 kcal/mol. Compounds **5**, **8,** and **9** showed potential *α*-glucosidase inhibitory activities, which were further confirmed by computational studies. The binding energies of compounds **5**, **8,** and **9** were lower than that of arcabose.

## 1. Introduction

Propolis is a sticky resinous substance that stingless bees, or honeybees, *Apis mellifera*, collect from various plants and trees to use in their hives. This bee product, alongside honey, holds significant medicinal value due to its chemical composition and biological activities [1,2]. Propolis is well known for its medicinal properties; it possesses a wide range of biological and pharmacological effects. It shows antibacterial, antioxidant, antitumor, immunostimulating, and antiviral activities, making it an important natural remedy for enhancing health and preventing diseases [1,2]. Recently, the scientists’ attention to stingless bee propolis has been growing, especially in Australia and the Indo-Malay peninsula due to the diverse species of stingless bees in these regions [3]. Previous chemical studies of stingless bee propolis in Australia and Southeast Asian countries led to the isolation of diverse chemical compounds, including triterpenes, flavonoids, coumarins, and xanthones [4,5,6,7,8,9,10,11,12,13,14,15,16,17,18,19,20].

*Homotrigona apicalis* (synonyms: *Trigona apicalis*, *Tetrigona apicalis*, and *Trigona hemileuca*) is a species of stingless bee found in Southeast Asia, particularly in countries such as Malaysia, Thailand, Indonesia, and Vietnam [3]. However, only a few studies of the chemical composition and biological activities of *H. apicalis/Tetrigona apicalis* propolis, originating from Malaysia and Vietnam, have been published. Omaz et al. reported that the EtOH extract of *T. apicalis* propolis showed weak antioxidant activity, with IC_50_ values of 2354.54 µg/mL for DPPH and 766.62 µg/mL for ABTS. The total phenolic and flavonoid contents of the propolis were determined as 28.57 µM gallic acid equivalent/g dry weight and 135.93 µM quercetin equivalent/g dry weight, respectively [21]. Mohamad et al. reported on the GC/MS profile, and high amounts of *α*- and *β*-amyrins and sesquiterpenoids in an underivatized 80% ethanol extract were found. The ethanol extract of *T. apicalis* propolis showed cytotoxicity against the MCF7 and MCF10 cancer cell lines (IC_50_ values ranging from 32 to 72 µg/mL) and possessed antioxidant activity against the ABTS+ radical with an IC_50_ value of 1.68 mg/mL [22]. In addition, in 2022 Mohamad’s group identified several compounds in *T. apicalis* propolis extract using Q-TOF LC-MS and evaluated the antioxidant effects of propolis, together with the total phenolic and flavonoid contents [23]. In addition to amino acid derivatives, only several terpenes, like (*S*)-*β*-himachalene, ishwarol, and oleanolic acid 3-O-*β*-D-glucosiduronic acid, were detected [23]. Recently, we analyzed propolis from *H. apicalis* from Vietnam, and triterpenes of the amyrin type were identified by GC-MS after derivatization [24].

Until now, however, there have been no investigations that have focused on the isolation of chemical compounds from *H. apicalis* propolis. In this study, we aimed to study the antimicrobial, cytotoxic, and *α*-glucosidase inhibitory activities of the EtOH extract and the isolated compounds from an *H. apicalis* propolis sample collected in Binh Dinh province. The interactions of several bioactive compounds with *α*-glucosidase and the cancer targets EGFR and HER2 were explored using a molecular docking study.

## 2. Materials and Methods

### 2.1. General Experimental Procedures

The ^1^H, ^13^C, HSQC, and HMBC NMR spectra were obtained using the Bruker AVANCE III HD 500 MHz and Bruker AVANCE NEO 600 MHz spectrometers (Bruker, Billerica, MA, USA); the chemical shift (*δ*) was expressed in ppm using TMS as an internal standard. The ESI-MS spectra were obtained using an Agilent 1260 series single quadrupole LC/MS system (Agilent, Santa Clara, CA, USA). Column chromatography (CC) was carried out on silica gel (Merck, Damstadt, Germany 40–63 μm) or Sephadex^®^ LH-20 (Sigma, Uppsala, Sweden). Analytical thin-layer chromatography was conducted on TLC aluminum sheet silica gel 60 F_254_ (Merck, Damstadt, Germany). The compounds were detected using a UV lamp (254 and 365 nm) or by spraying with 10% sulfuric acid in water and heating.

### 2.2. Propolis Material

The propolis sample was collected from a stingless bee hive in Hoai Nhon district, Binh Dinh province, Vietnam, in March 2022. The stingless bee species was identified as *Homotrigona apicalis* (Smith, 1857) by Prof. Nguyen Thi Phuong Lien and M.Sc Tran Thi Ngat of the Institute of Ecology and Biological Resources, Vietnam Academy of Science and Technology.

### 2.3. Extraction and Isolation

The propolis sample (500 g) was extracted with 70% EtOH (4 × 5 L for 24 h) at room temperature. The EtOH extracts were combined and evaporated under reduced pressure.

The obtained EtOH extract was suspended in distilled water and extracted with ethyl acetate (EtOAc) to give EtOAc residue (215 g). The EtOAc residue was chromatographed on silica gel CC, using a solvent gradient of *n*-hexane/EtOAc as an eluent, to afford ten fractions (E1–E10). Fraction E2 (5 g) was fractionated by silica gel CC eluting with *n*-hexane/acetone (19:1) to yield ten fractions E2.1–E2.10. Fraction E2.2 (98 mg) was separated by silica gel CC and eluted with *n*-hexane/EtOAc (19:1) to yield **1** (4.1 mg). Fraction E2.10 (54 mg) was purified by silica gel CC eluting with *n*-hexane/acetone (9:1) to yield **5** (5.4 mg).

Fraction E5 (5.3 g) was subjected to silica gel CC and eluted with *n*-hexane/EtOAc (9:1) to afford eight fractions, E5.1–E5.8. Fraction E5.3 (3.2 g) was separated by silica gel CC and eluted with *n*-hexane/acetone (9:1) to afford six fractions, E5.3.1–E5.3.6. Fraction E5.3.2 (112 mg) was purified by silica gel CC, using *n*-hexane/acetone (8:2) as a mobile phase, to yield **8** (3.5 mg). Fraction E5.6 (155 mg) was purified by silica gel CC eluting with *n*-hexane/EtOAc (9:1) to yield **6** (5.5 mg).

Fraction E7 (10 g) was chromatographed on silica gel CC using *n*-hexane/EtOAc (8:2) as an eluent to give eight fractions, E7.1–E7.8. Compound 7 (50 mg) was obtained from fraction E7.7. Fraction E7.3 was separated by silica gel CC and eluted with *n*-hexane/EtOAc (8:2) to give eight sub-fractions, E7.3.1–E7.3.8. Fraction E7.3 4 (144 mg) was separated by reversed phase silica gel CC and eluted with MeOH/water (2:1) to give **3** (5 mg). Sub-fraction E7.8 (0.5 g) was purified by Sephadex^®^ LH-20 CC and eluted with MeOH/CH_2_Cl_2_ (9:1) to give two sub-fractions, E7.8.1–E7.8.2. Sub-fraction E7.8.2 (50 mg) was purified by Sephadex^®^ LH-20 CC and eluted with MeOH to give compound **9** (4.5 mg).

Fraction E9 (5.3 g) was separated on silica gel CC and eluted with *n*-hexane/EtOAc (7:3) to give nine fractions, E9.1–E9.9. Fraction E9.7 (415 mg) was separated by reversed phase silica gel CC and eluted with MeOH/water (1:1) to give six fractions, E.9.7.1–E.9.7.6. Fraction E9.7.6 (62 mg) was purified by Sephadex^®^ LH-20 CC and eluted with MeOH to give compound **2** (4.5 mg). Fraction E9.8 (486 mg) was separated by reversed-phase silica gel CC and eluted with MeOH-water (2:1) to give ten fractions, E.9.8.1–E.9.8.10. Fraction E9.8.2 (99 mg) was purified by silica gel CC using *n*-hexane/EtOAc (6:4) as an eluent to afford compound **4** (5.6 mg).

### 2.4. Antimicrobial Activity

The antimicrobial activity assay was performed using the broth microdilution method [24]. Dimethyl sulfoxide (DMSO) was used for the preparation of compound solutions at concentrations of 256 μg/mL, 64 μg/mL, 16 μg/mL, 4 μg/mL, and 1 μg/mL. Three strains of Gram-positive bacteria (*Staphylococcus aureus*, *Bacillus cereus*, and *Lactobacillus fermentum)*; three strains of Gram-negative bacteria (*Salmonella enterica*, *Escherichia coli*, and *Pseudomonas aeruginosa*), and fungus *Candida albicans* (obtained from the American Type Culture Collection (ATCC)) were used for the test. Ampicillin, Cefotaxim, and Nystatin were used as positive controls for the Gram (+) bacterial and Gram (−) bacterial strains and *C. albicans*, respectively. The activity of the isolated compounds was expressed as an IC_50_ value (half-maximal inhibitory concentration). The IC_50_ values were calculated using the program Raw Data.

### 2.5. Cytotoxicity Assay

The cytotoxic assays were evaluated in triplicate against three cancer cell lines, namely KB (epidermal carcinoma), A-549 (human lung adenocarcinoma), and HepG-2 (human hepatoma) (ATCC), with a modification of the previously published method [25]. The cells were cultured in Dulbecco’s D-MEM medium (Gibco, Life Technologies Corporation, Grand Island, NY, USA), supplemented with 10% fetal calf serum (Sigma-Aldrich, St. Loius, MO, USA), L-glutamine (2 mM) (Sigma-Aldrich, St. Loius, MO, USA), penicillin G (100 UI/mL), and streptomycin (100 μg/mL) (Sigma-Aldrich, St. Loius, MO, USA). The compound solutions were prepared in DMSO, and the cytotoxic assays were conducted in 96-well microtiter plates against the cancer cells (3 × 10^5^ cells/mL concentration). After 48 h incubation at 37 °C in an air/CO_2_ (95:5) atmosphere, with or without isolated compounds, MTT reagent was added to each well (0.5 mg/mL). The cell growth was assessed by measuring the colorimetric of formazan at 570 nm using a microplate reader. The IC_50_ value that gave the concentration of a sample necessary to inhibit the cell growth to 50% of the control was determined. Ellipticine was used as a positive reference.

### 2.6. *α*-Glucosidase Inhibitory Activity

An assay for the *α*-glucosidase inhibitory activity was carried out by a modification of the published method [26]. Briefly, solutions of *α*-glucosidase (*Saccharomyces cerevisiae*, G0660, Sigma-Aldrich, St. Louis, MO, USA) and *p*-nitrophenyl *α*-D-glucopyranoside (Sigma-Aldrich, St. Louis, MO, USA) were prepared in a phosphate buffer (100 mM, pH 6.8, Sigma-Aldrich, St. Louis, MO, USA). Solutions of isolated compounds at concentrations of 128 μg/mL, 32 μg/mL, 8 μg/mL, and 2 μg/mL were prepared using DMSO (Sigma-Aldrich, St. Louis, MO, USA). The compound solution (2 μL) and 40 µL of enzyme solution (0.5 U/mL) in 120 µL phosphate buffer were mixed. After 5 min of preincubation, 20 µL of 10 mM *p*-nitrophenyl *α*-D-glucopyranoside prepared in phosphate buffer was added, and the reaction mixture was incubated for 30 min at 37 °C. The reaction was quenched by the addition of 80 µL of 0.2 M Na_2_CO_3_. The absorbance of the released *p*-nitrophenol was measured at 410 nm using a Biotek reader. The % inhibition was calculated using the following equation:

Inhibition (%) = [1 − (A_sample_/A_control_)] ∗ 100

Acarbose was used as a reference compound. The IC_50_ values of the isolated compounds were calculated using the program Table Curve.

### 2.7. Molecular Docking

In this study, molecular docking was carried out to further investigate the binding modes of the isolated compounds to *α*-glucosidase, as well as to some of the targets associated with the cytotoxic activities of these compounds. For the cytotoxic effects, two proteins were selected: EGFR and HER2 [27]. The structures of these two targets were obtained from the protein databank, RCSB, with the respective IDs of 4HJO and 3PP0. As the structure of *α*-glucosidase of *Saccharomyces cerevisiae* was not available in the RCSB database, a homology model was employed using the isomaltase of *S. cerevisiae* as the template (PDB ID: 3AJ7). In this study, the homology model was generated and evaluated using the Swiss-Model server, following the protocol described by Nguyen Ngoc Tuan et al. [28]. After removing the water molecules, hydrogen atoms were added, and partial charges were assigned using Autodock Tool 1.5.6 software. Subsequently, the protein structures were saved in a pdbqt format.

The ligand structures were obtained from the PubChem database or generated using BIOVIA Discovery Studio Visualizer 4.5 and then converted to pdbqt files. Autodock Vina software was used to perform molecular docking between the isolated compounds and the selected targets. In this study, the size of the grid box was set as 20 × 20 × 20 Å^3^ for EGFR and HER2 and 25 × 25 × 25 Å^3^ for *α*-glucosidase. The coordinates for the grid box center were as follows: x = 24.135, y = 9.847, and z = 0.741 for EGFR; x = 15.809, y = 16.895, and z = 27.075 for HER2; and x = 20.226, y = −8.148, and z = 17.909 for *α*-glucosidase. The exhaustiveness parameter was set to 8. The docking results were analyzed using BIOVIA Discovery Studio Visualizer 4.5.

In this study, the three positive controls corresponding to the three enzymes (EGFR, HER2, and *α*-glucosidase) were erlotinib, 2-{2-[4-({5-chloro-6-[3-trifluoromethyl)phenoxy] pyridin-3-yl}amino)-5H-pyrrolo [3,2-d]pyrimidin-5-yl]ethoxy}ethanol (03Q), and acarbose, respectively. Additionally, to confirm the accuracy of the docking protocol used in the study, the cocrystal ligands of EGFR (erlotinib) and HER2 (03Q) were re-docked and compared with the experimental results based on the root mean square deviation (RMSD) values. The docking protocol was considered accurate if the RMSD value was less than 2.0 Å [29].

## 3. Results and Discussion

### 3.1. Isolation and Compound Identification

**Figure 1:**
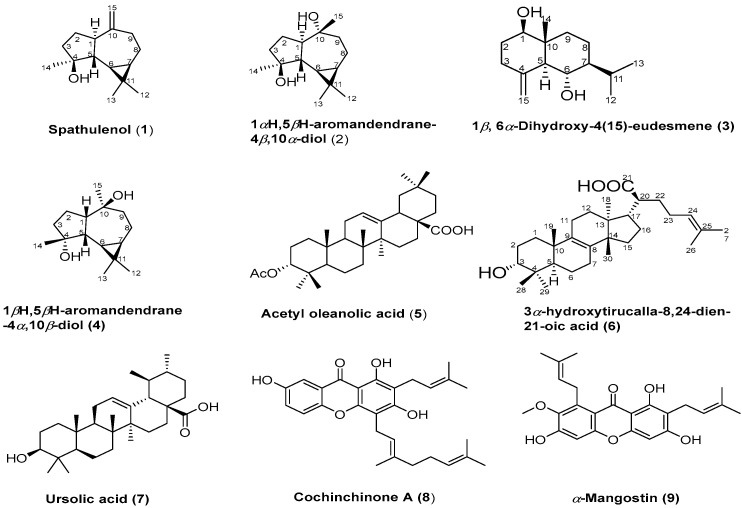
Chemical structures of isolated compounds.

In this study, we report for the first time the characterization of individual compounds of the *H. apicalis* propolis. From a propolis sample collected in Binh Dinh province, a total of nine constituents were isolated (Figure 1), including four sesquiterpenes: spathulenol (**1**), 1*α*H,5*β*H-aromandendrane-4*β*,10*α*-diol (**2**), 1*β*,6*α*-dihydroxy-4(15)-eudesmene (**3**), and 1*β*H,5*β*H-aromandendrane-4*α*,10*β*-diol (**4**); three triterpenes: acetyl oleanolic acid (**5**), 3*α*-hydroxytirucalla-8,24-dien-21-oic acid (**6**), and ursolic acid (**7**); and two xanthones: cochinchinone A (**8**) and *α*-mangostin (**9**). The chemical structures of the isolated compounds were elucidated by NMR and by the comparison of the data (SI) with those in the literature [5,30,31,32,33,34,35,36,37,38]. Xanthones **8** and **9** were previously isolated from *Lisotrigona* spp. Propolis from Vietnam [12,15], while triterpenes **5** and **7** were characterized from *Tetrigona melanolauca* propolis collected in Thailand [5]. Sesquiterpens **1**–**4** and triterpene **6** from the propolis of stingless bees were reported for the first time.

Xanthones **8** and **9** have been recognized as major secondary metabolites of *Cratoxylum cochinchinense* [37,38], and both compounds were previously isolated from *Lisotrigona* spp. Propolis in the same region [12,15]. Hence, *C. cochinchinense* was identified as one of the plant sources of *H. apicalis* propolis in Binh Dinh province.

The presence of sesquiterpenes **1**–**4** and triterpene **7** suggested that *Aglaia* species (Meliaceae) might be plant sources of the analyzed propolis. Spathulenol (**1**) and 1*α*H,5*β*H-aromandendrane-4*β*,10*α*-diol (**2**) were concurrently isolated from the stem bark of *Aglaia harmsiana* [39]. Spathulenol (**1**), 1*β*,6*α*-dihydroxy-4(15)-eudesmene (**3**), and ursolic acid (**7**) were found in the stembark of *Aglaia foveolate* and *A. forbesii*, *A. minahasae*, and *A. duperreana*, respectively [40,41,42,43,44]. In Vietnam, there are around thirty *Aglaia* species [45,46]; among them, *Aglaia odorata* [47], *Aglaia macrocarpa*, and *A. eximia* [45] are widely found in Binh Dinh province, where the *H. apicalis* propolis was collected. Nevertheless, the participation of some dipterocarp trees is also suggested to be a possible source for the propolis production as the resin they produce is characterized by a combination of sesquiterpenes and triterpenes, mainly of the amyrin type [24].

### 3.2. Antibacterial Activity

The EtOH extract and the isolated compounds were evaluated for their antimicrobial activity. As shown in the Table 1, the EtOH extract showed moderate activity against *S. aureus* and was not active against the other bacterial strains and *C. albicans* at a 256 µg/mL concentration. However, among the isolated compounds, ursolic acid (**7**), *α*-mangostin (**8**), and cochinchinone A (**9**) showed good to strong antibacterial activity on the Gram (+) bacterial strains. The antibacterial activities of *α*-mangostin (**8**) and cochinchinone A (**9**) were also reported in our previous studies of stingless bee propolis [12,15]. In our antimicrobial activity assay, ursolic acid also exhibited good antibacterial activity against *S. aureus*, *B. cereus*, and *L. fermentum*, with IC_50_ values of 7.10 ± 0.25, 10.19 ± 0.67, and 10.61 ± 0.70 µg/mL, respectively.

### 3.3. Cytotoxic Activity

The EtOH extract and isolated compounds were assayed for their cytotoxicity against KB, A-549, and HepG2 cancer cell lines using the MTT method. As shown in Table 2, the EtOH extract displayed selective activity against the A-549 cancer cell line, with IC_50_ values of 22.82 ± 0.86 µg/mL, 61.29 ± 1.58 µg/mL, and 124.91 ± 3.05 µg/mL for the A-549, HepG-2, and KB cancer cell lines, respectively.

Among the isolated compounds, xanthones **8** and **9** showed good cytotoxicity against all the tested cancer cell lines, with IC_50_ values ranging from 7.55 ± 0.25 to 29.27 ± 2.07 µg/mL; these values were in agreement with those of our previous study [15]. Compound **6** exhibited moderate cytotoxicity on the tested cancer cell lines, while the other compound was not active at a 128 µg/mL concentration.

### 3.4. *α*-Glucosidase Inhibitory Activity

The *α*-Glucosidase inhibitory activities of the EtOH extract and the isolated compounds were evaluated. The EtOH extract showed similar *α*-glucosidase inhibitory activities to that of the reference compound arcabose (IC_50_ value of 134.56 ± 3.02 μg/mL). In the assay, 3-acetyl oleanolic (**5**), *α*-mangostin (**8**), and cochinchinone A (**9**) showed strong *α*-glucosidase inhibitory activities, with IC_50_ values ranging between 1.85 ± 0.12 μg/mL and 9.07 ± 0.65 μg/mL. Ursolic acid exerted moderate enzyme inhibitory activity with an IC_50_ value of 25.34 ± 0.54 μg/mL. The *α*-glucosidase inhibitory activities of these compounds were in agreement with those of several previous studies [47,48,49].

### 3.5. Molecular Docking

The results from Table 2 revealed that xanthones **8** and **9** exhibited the strongest cytotoxic effects on all three cell lines: KB, A-549, and Hep-G2. Consequently, these two compounds were selected for molecular docking with the potential cancer targets. In this study, the chosen targets were EGFR and HER. The epidermal growth factor receptor (EGFR) and human epidermal growth factor receptor 2 (HER2) have been extensively studied in the context of anticancer drug discovery [50]. Both EGFR and HER2 belong to the receptor tyrosine kinase family and are frequently implicated in various cancer types [51]. Their signaling pathways play crucial roles in promoting cell proliferation, survival, angiogenesis, and metastasis, rendering them attractive targets for therapeutic intervention. In many cancer types, the mutations, overexpression, or amplification of EGFR or HER2 contribute to tumorigenesis and are associated with aggressive tumor behavior and poor patient prognosis. Consequently, drugs targeting EGFR and HER2 have demonstrated remarkable clinical efficacy in diverse malignancies, including breast, lung, and gastrointestinal cancers [52,53]. Moreover, the combination of EGFR- and HER2-targeted agents has shown synergistic effects in specific tumor subtypes [27]. Hence, in this study, EGFR and HER2 were selected to investigate the interaction between **8** and **9**, the two isolated potential compounds.

**Figure 2:**
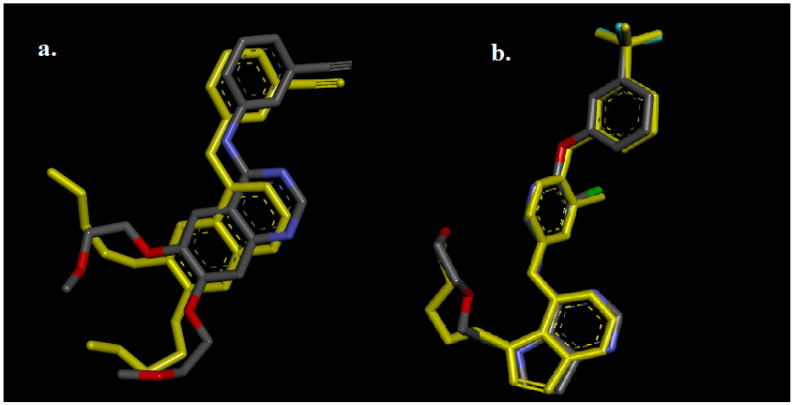
Superimposed image of the cocrystal ligands: erlotinib (**a**) and 03Q (**b**) and the redocked ligands (yellow).

Before carrying out the molecular docking, the protocol was validated by redocking the cocrystal ligands into the active site of the enzymes and comparing the results with experimental data based on the root mean square deviation (RMSD) value. The results demonstrated the high accuracy of the protocol employed in this study, with the RMSD values for erlotinib and 03Q of 1.39 Å and 0.46 Å, respectively. As both values were below 2 Å, it is suggested that the molecular docking method could be effectively used to investigate the interactions between the potential compounds and their respective targets. Figure 2 illustrates the superimposed image of erlotinib with 03Q, along with the redocking results.

Using the validated molecular docking protocol mentioned earlier, the two compounds **8** and **9** were docked into the active sites of the enzymes EGFR and HER2. The resulting data are presented in Table 3.

**Figure 3:**
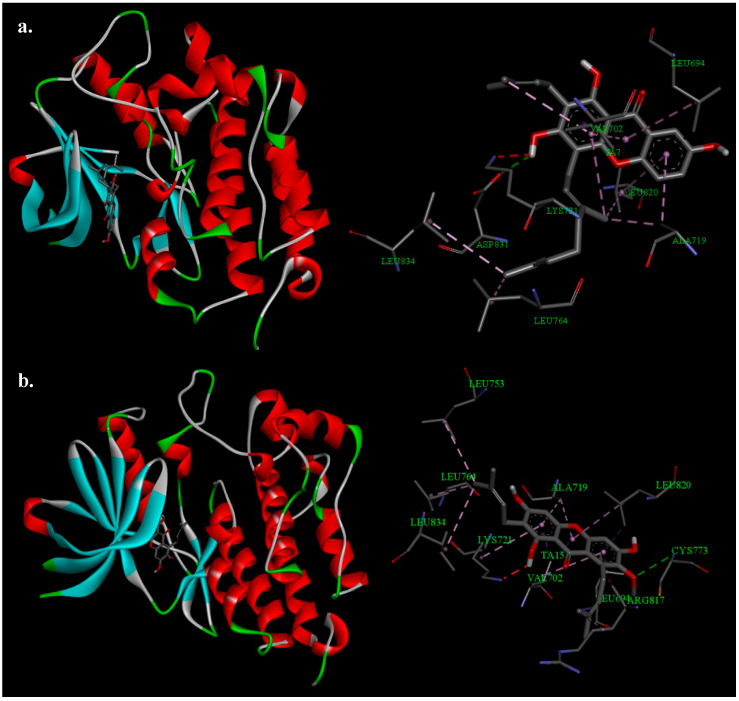
Binding mode of EGFR with xanthones **8** (**a**) and **9** (**b**).

As can be seen in Table 3, both compounds (**8** and **9**) exhibited lower binding scores compared to erlotinib, a clinically used EGFR inhibitor. Specifically, the binding affinities of **8** and **9** were −9.7 and −9.3 kcal/mol, respectively, whereas erlotinib had a binding affinity of −7.2 kcal/mol. These results were also consistent with the in vitro experiment where **8** and **9** demonstrated strong cytotoxic effects on all three cell lines (KB, A-549, and Hep-G2), with IC_50_ values ranging from 7.55 ± 0.25 to 29.27 ± 2.07 μg/mL. Further analysis of the interaction between these potential compounds and EGFR (Figure 3) revealed a similarity in the binding modes between these two compounds and the enzyme. Notably, the key residues involved in the interaction included Leu694, Val702, Ala719, Lys721, Leu764, Leu820, and Leu834. These residues might play an important role in the activities of EGFR as well as in the research and development of EGFR inhibitors.

**Figure 4:**
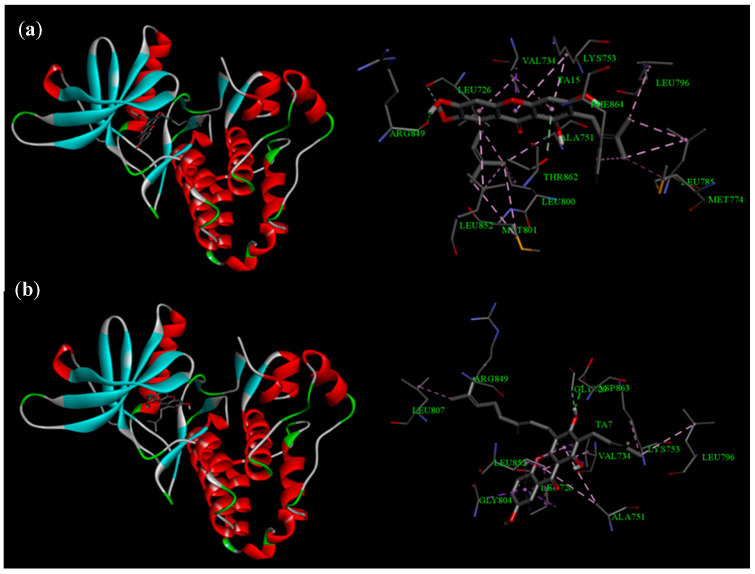
Binding mode of HER2 with xanthones **8** (**a**) and **9** (**b**).

Regarding HER2, while the affinity of **8** and **9** was not lower than that of the positive control (03Q), their low docking scores (−9.9 kcal/mol for TA7 and −9.7 kcal/mol for TA15) still indicated a strong potential to bind with HER2. The analysis of the binding modes between **8** and **9** with HER2 (Figure 4) also yielded similar results, with 11 and 14 amino acid residues involved in the interactions, respectively. Among the identified residues, Gly804, Leu726, Leu852, Val734, Ala751, Arg849, and Lys753 were found to be of particular importance. Based on the molecular docking results, it is suggested that one of the mechanisms underlying the cytotoxic effects of **8** and **9** functions through the inhibition of the EGFR and HER2 pathways.

Regarding the inhibitory effect of the isolated compounds on *α*-glucosidase, three potential compounds, **5**, **8**, and **9,** were selected for further analysis based on the in vitro results. Acarbose, a well-known *α*-glucosidase inhibitor, was used as the positive control. The results revealed that all the other compounds exhibited lower binding energies than acarbose. Notably, **8** and **9** demonstrated excellent binding capacity, with docking scores of −9.3 kcal/mol and −9.9 kcal/mol, respectively, compared to acarbose’s affinity of −7.6 kcal/mol (Table 3). These findings were consistent with the in vitro experiment, where **8** and **9** displayed significantly lower IC_50_ values than that of acarbose. Specifically, **8** and **9** exhibited IC_50_ values of 9.07 ± 0.65 μg/mL and 1.90 ± 0.10 μg/mL, respectively, while the IC_50_ of acarbose was 134.56 ± 3.02 μg/mL.

**Figure 5:**
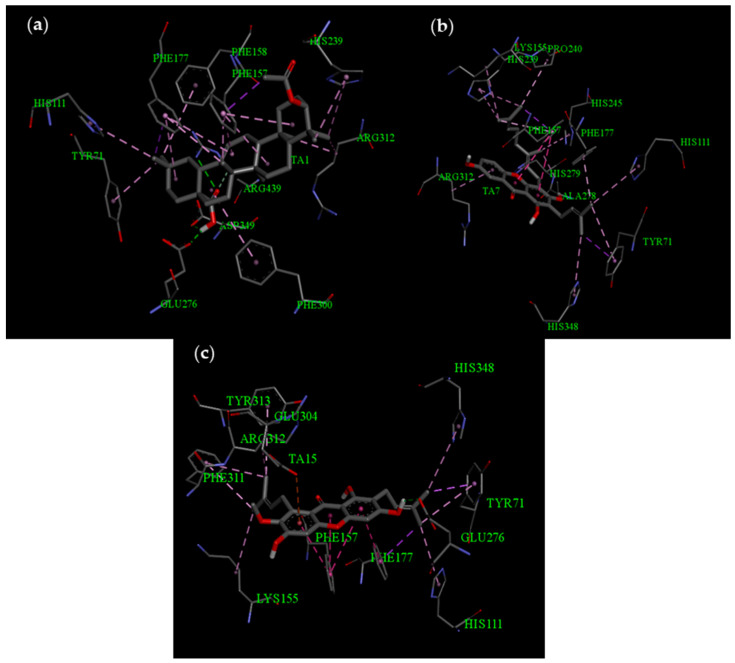
Binding mode of *α*-glucosidase with **5** (**a**), **8** (**b**), and **9** (**c**).

The analysis of the binding modes of **8** and **9** highlighted the important residues involved in the interaction, including Tyr71, His348, Arg312, Phe157, Lys155, Phe177, and His111 (Figure 5). For compound **5**, although its binding energies were equivalent to that of acarbose, the in vitro results revealed a strong *α*-glucosidase inhibition, with IC_50_ values of 8.44 ± 0.23 μg/mL. The binding mode of **5** with *α*-glucosidase revealed that this compound still exhibited strong interactions with important residues such as Arg312, Phe177, Tyr71, Phe158, Phe300, Asp349, Arg439, His111, Phe157, and His239. These interactions may be the reasons for the good *α*-glucosidase inhibitory activities of this substance.

To the best of our knowledge, this is the first study to investigate the interaction between cochinchinone A (**8**) and EGFR, HER2, and *α*-glucosidase. Regarding α-mangostin, several previous studies performed molecular docking to examine the binding mode of this compound to *α*-glucosidase; however, these studies still have certain limitations. For example, Faisal Usman et al. [54], Qirou Wang et al. [55], and Juan Cardozo-Muñoz et al. [56] conducted computational experiments without a positive control, resulting in insufficient evidence to support the reliability of their docking results for α-mangostin and *α*-glucosidase. On the other hand, Nina Artanti et al. [57] and Ahmad Fariz Maulana et al. [58] used acarbose as a positive control, but their docking results did not accurately reflect the in vitro experimental results. In this study, α-mangostin was demonstrated to be a potent *α*-glucosidase inhibitor, with an IC_50_ value of 1.90 ± 0.10 μg/mL (equivalent to 4.63 ± 0.24 μM), whereas the IC_50_ value of acarbose was 134.56 ± 3.02 μg/mL (equivalent to 204.93 ± 4.60 μM). These results are consistent with a previous study conducted by Nam K. Nguyen et al., which reported IC_50_ values of 11.4 ± 2.3 μM for α-mangostin and 214.5 ± 2.3 μM for acarbose [59]; they are also consistent with the findings of H. W. Ryu et al., who reported an IC_50_ value of 5.0 ± 0.1 μM for α-mangostin [60]. However, in the molecular docking studies performed by Nina Artanti, the docking scores for acarbose and α-mangostin were −92.3 ± 2.2 and −73.4 ± 2.0, respectively. Similarly, Ahmad Fariz Maulana’s study revealed binding affinities of −7.1 and −7.3 kcal/mol for these two compounds, respectively. These results failed to explain the significant disparity in the *α*-glucosidase inhibitory activity between α-mangostin and acarbose observed in in vitro experiments. In contrast, our study consistently demonstrates a substantial difference in binding energies between acarbose and α-mangostin with *α*-glucosidase, as evidenced by the molecular docking affinity scores of −7.6 and −9.9 kcal/mol, respectively. Moreover, previous studies exploring the structure–activity relationship of *α*-glucosidase inhibitors identified crucial residues involved in the pharmacological effects of this enzyme, including Tyr71, Glu276, His348, and Asp408 [61]; these findings are also consistent with our molecular docking results for *α*-glucosidase and acarbose, as well as compounds **5**, **8**, and **9** (Table 3). These findings once again demonstrate the reliability and accuracy of our results.

## 4. Conclusions

The EtOH extract and isolated compounds of *Homotrigona apicalis* propolis collected in Binh Dinh province, Vietnam, were evaluated for antibacterial, cytotoxic, and *α*-glucosidase inhibitory activities. The chromatographic purification led to the isolation of nine compounds, **1**–**9**, among which **1**–**4** and **6** were found for the first time in stingless bee propolis. *Cratoxylum* and *Aglaia* sp. were suggested as probable plant sources of the propolis sample. In the antibacterial activity evaluation, the EtOH extract only showed moderate activity on *S. aureus*, while compounds **7**–**9** were found to possess good antibacterial activity, with IC_50_ values of 0.56 to 17.33 µg/mL. The EtOH extract displayed selective cytotoxicity against the A-549 cancer cell line, with IC_50_ values of 22.82 ± 0.86 µg/mL, while xanthones **8** and **9** were active against the A-549, KB, and HepG-2 cancer cell lines, with IC_50_ values ranging from 7.55 ± 0.25 to 29.27 ± 2.07 µg/mL. The cytotoxic effects of xanthone **8** and **9** were determined by the inhibition of the EGFR and HER2 pathways using a molecular docking study. Compounds **5**, **8**, and **9** showed potential *α*-glucosidase inhibitory effects; these effects were in agreement with the computational experiments.

## Figures and Tables

**Table 1:** Antimicrobial activity of EtOH extract and isolated compounds.

Compound	IC_50_ (µg/mL)						
	Gram (+) Strains			Gram (−) Strains			Fungus
	*S. aureus*	*B. cereus*	*L. fermentum*	*S. enterica*	*E. coli*	*P. aeruginosa*	*C. albicans*
**1**	>256	>256	205.71 ± 5.23	>256	>256	>256	>256
**2**	>256	>256	>256	>256	>256	>256	>256
**3**	>256	>256	>256	>256	>256	>256	>256
**4**	>256	>256	>256	>256	>256	>256	>256
**5**	>256	>256	49.88 ± 1.06	>256	>256	>256	>256
**6**	>256	140.8 ± 4.14	62.22 ± 1.24	>256	>256	>256	>256
**7**	7.10 ± 0.25	10.19 ± 0.67	10.61 ± 0.70	>256	>256	>256	>256
**8**	17.33 ± 0.87	7.99 ± 0.33	8.67 ± 0.38	>256	>256	35.44 ± 2.03	65.33 ± 3.54
**9**	0.54 ± 0.07	0.57 ± 0.07	0.56 ± 0.09	16.33 ± 0.78	7.89 ± 0.34	1.22 ± 0.09	0.56 ± 0.06
EtOH extract	133.1 ± 4.1	>256	>256	>256	>256	>256	>256
A	0.02 ± 0.005	3.62 ± 0.15	1.03 ± 0.07				
C				0.43 ± 0.05	0.07 ± 0.02	4.34 ± 0.15	
N	-	-	-	-	-	-	1.32 ± 0.05

**Table 2:** Cytotoxicity and *α*-glucosidase inhibitory activity of isolated compounds.

Compound	IC_50_ (µg/mL)			
	KB	A-549	Hep-G2	*α*-Glucosidase Inhibitory Activity
**1**	>128	>128	>128	>256
**2**	>128	>128	>128	>256
**3**	>128	>128	>128	>256
**4**	89.10 ± 9.67	>128	>128	>256
**5**	>128	>128	>128	8.44 ± 0.23
**6**	49.31 ± 5.21	78.63 ± 7.69	77.71 ± 8.09	>256
**7**	>128	>128	>128	25.34 ± 0.54
**8**	29.27 ± 2.07	20.33 ± 1.34	17.36 ± 1.03	9.07 ± 0.65
**9**	9.28 ± 0.58	7.55 ± 0.25	12.90 ± 0.68	1.90 ± 0.10
EtOH extract	124.91 ± 3.05	22.82 ± 0.86	61.29 ± 1.58	146.33 ± 5.04
Ellipticine	0.48 ± 0.03	0.34 ± 0.02	0.50 ± 0.03	
Arcabose				134.56 ± 3.02

**Table 3:** Molecular docking results between isolated compounds and EGFR, HER2, *α*-glucosidase.

Compound	Target	Docking Score (kcal/mol)	Interacted Residues
**8**	EGFR	−9.7	Leu694, Val702, Ala719, Lys721, Leu764, Leu820, Asp831, Leu834
**9**		−9.3	Leu694, Val702, Ala719, Lys721, Leu753, Leu764, Cys773, Arg817, Leu820, Leu834
Erlotinib		−7.2	Leu694, Val702, Ala719, Lys721, Leu764, Gln767, Met769, Leu820, Thr830
**8**	HER2	−9.9	Gly804, Leu726, Leu852, Val734, Ala751, Arg849, Leu807, Gly729, Asp863, Lys753, Leu796
**9**		−9.7	Val734, Lys753, Thr862, Leu785, Met774, Phe864, Leu796, Met801, Gly804, Ala751, Leu800, Leu852, Leu726, Arg849
03Q		−11.2	Leu852, Leu726, Gly739, Asn850, Thr862, Val734, Leu785, Glu770, Met774, Leu796, Phe864, Lys753, Ala751, Gln799, Met801
**5**	*α*-glucosidase	−7.9	Arg312, Phe177, Tyr71, Phe158, Phe300, Asp349, Arg439, His111, Phe157, His239
**8**		−9.3	Tyr71, His348, Arg312, Phe157, Pro240, Lys155, His239, His245, His279, Ala278, Phe177, His111
**9**		−9.9	Tyr71, His111, His348, Phe177, Glu276, Phe157, Glu204, Arg312, Phe311, Tyr313, Lys155
Acarbose		−7.6	Glu304, His279, Pro309, Phe300, Arg312, Glu276, Gln350, Asp349, Tyr313, Asp408, Phe157

## Data Availability

The data presented in this study are available upon request from the corresponding author. Data is contained within the article and Supplementary Material.

## Supplementary Materials

The following supporting information can be downloaded at: https://www.mdpi.com/article/10.3390/life13081682/s1, Figures S1–S28: Physical and Spectroscopic and Spectral Data of Compounds **1**–**9**, Table S1: Docking scores of isolated compounds with *α*-glucosidase.

## Sample Availability

Samples are available upon reasonable request from the corresponding author.
