# Supplementary material for: Antimicrobial, Cytotoxic, and α-Glucosidase Inhibitory Activities of Ethanol Extract and Chemical Constituents Isolated from Homotrigona apicalis Propolis—In Vitro and Molecular Docking Studies
Source: Life (Basel). 2023 Aug 3;13(8):1682. doi: 10.3390/life13081682 (PMC10455239; doi:10.3390/life13081682)
Supplement: Supplementary file 1 [file life-13-01682-s001.zip › SI H.apicalis.pdf]

SUPPLEMENTARY MATERIAL

Antimicrobial, cytotoxic and α-glucosidase inhibitory activities of constituents isolated from *Homotrigona apicalis* propolis - *In vitro* and molecular docking studies

Diep Thi Lan Phuong[1,*], Nguyen Van Phuong[2], Nguyen Le Tuan[1], Nguyen Thanh Cong[2,3], Nguyen Thu Hang[2], Le Nguyen Thanh[4,5,*], Vu Thi Hue[5], Nguyen Quoc Vuong[4,5], Nguyen Thi Thu Ha[6], Milena Popova[7], Boryana Trusheva[7], Vassya Bankova[7]

[1] *Faculty of Natural Sciences, Quy Nhon University, Binh Dinh, Vietnam*

[2] *Hanoi University of Pharmacy, Hanoi, Vietnam*

[3] *Department of Pharmacy, Dai Nam University, Hanoi, Vietnam*

[4] *Graduate University of Science and Technology, Vietnam Academy of Science and Technology (VAST), Hanoi, Vietnam*

[5] *Institute of Marine Biochemistry, VAST, Hanoi, Vietnam*

[6] *Institute of Chemistry, VAST, Hanoi, Vietnam*

[7] Institute of Organic Chemistry with Centre of Phytochemistry, Bulgarian Academy of Sciences, Acad. G. Bonchev str., bl.9, Sofia, Bulgaria

4

**Correspondence:* diepthilanphuong@qnu.edu.vn *(Diep Thi Lan Phuong)*

lethanh@imbc.vast.vn (*Le Nguyen Thanh)*

ABSTRACT

The chemical investigation of *Homotrigona apicalis* propolis collected in Binh Dinh province, Vietnam lead to the isolation of nine compounds, including four sesquiterpenes: spathulenol (**1**), 1*α*H,5*β*H-aromandendrane-4*β*,10*α*-diol (**2**), 1*β*, 6*α*-Dihydroxy-4(15)-eudesmene (**3**) and 1*β*H,5*β*H-aromandendrane-4*α*,10*β*-diol (**4**), three triterpenes: acetyl oleanolic acid (**5**), 3*α*-hydroxytirucalla-8,24-dien-21-oic acid (**6**) and ursolic acid (**7**), two xanthones: cochinchinone A (**8**) and *α*-mangostin (**9**). Sesquiterpens **1–4** and triterpene **6** were isolated for the first time from stingless bee propolis. In the antibacterial activity evaluation, the EtOH extract only showed moderate activity on *S. aureus* but isolated compounds **7-9** showed good antibacterial activity with IC_{50} values of 0.56 to 17.33 μg/mL. The EtOH extract displayed selective cytotoxicity against A-549 cancer cell line with IC_{50} values of 22.82±0.86 μg/mL, while xanthones **8** and **9** exhibited good activity against KB, HepG-2 and A-549 cancer cell lines with IC_{50} values range from 7.55 ± 0.25 to 29.27 ± 2.07 μg/mL. Cytotoxic effects of xanthone **8** and **9** are proposed by the inhibition of the EGFR and HER2 pathways using molecular docking study. Compounds **5**, **8** and **9** showed potential *α*-glucosidase inhibitory activities, that was further confirmed by computational studies.

Keywords: *Homotrigona apicalis;* sesquiterpenes; triterpenes; xanthones; *α*-glucosidase; cytotoxicity

Physical and Spectroscopic Data of Compounds

Spathulenol (**1**) Colorless oil; $[\alpha]_D^{25}$ + 50.3 (*c* 0.1, $CHCl_3$); ESI-MS *m/z* 219 $[M-H]^-$. ^{1}H-NMR (600 MHz, $CDCl_3$) δ (ppm): 4.69 (1H, br s, H-15a), 4.66 (1H, br s, H-15b), 2.42 (1H, dd, *J* = 6.6 Hz, 13.2 Hz, H-9b), 2.20 (1H, m, H-1), 2.05 (1H, m, H-9a), 1.97 (1H, m, H-8b), 1.92 (1H, m, H-2b), 1.78 (1H, m, H-3b), 1.64 (1H, m, H-2a), 1.54 (1H, m, H-3a), 1.31 (1H, t, *J* = 10.8 Hz, H-5), 1.28 (3H, s, H-14), 1.05 (3H, s, H-12), 1.04 (3H, s, H-13), 1.01 (H-8a), 0.71 (1H, m, H-7), 0.47 (1H, dd, *J* = 9.6, 11.4 Hz, H-6).
^{13}C NMR (150 MHz, $CDCl_3$) δ (ppm): 153.5 (C-10), 106.3 (C-15), 81.0 (C-4), 54.4 (C-5), 53.4 (C-1), 41.7 (C-3), 38.9 (C-9), 29.9 (C-6), 28.7 (C-12), 27.5 (C-7), 26.7 (C-2), 26.1 (C-14), 24.8 (C-8), 20.3 (C-11), 16.3 (C-13).

1*α*H,5*β*H-aromandendrane-4*β*,10*α*-diol (2) Colorless oil, ESI-MS *m/z* 237 $[M-H]^-$. ^{1}H-NMR (600 MHz, $CDCl_3$) δ (ppm): 1.86 (1H, m, H-1), 1.82 (1H, m, H-8b), 1.76 (1H, dd, J = 6.6 Hz, 13.2 Hz, H-9b), 1.69-1.58 (4H, m, H-2, H-3), 1.72 (1H, t, J = 13.2 Hz, H-9a), 1.28 (3H, s, H-14), 1.18 (1H, t, J = 10.8 Hz, H-5), 1.17 (3H, s, H-15), 1.06 (6H, s, H-12, H-13), 0.88 (1H, m, H-8a), 0.64 (1H, m, H-7), 0.43 (1H, dd, J = 9.6 Hz, 10.8 Hz, H-6). ^{13}C NMR (150 MHz, $CDCl_3$) δ (ppm): 80.4 (C-4), 75.0 (C-10), 56.4 (C-1), 48.5 (C-5), 44.5 (C-9), 41.2 (C-3), 28.7 (C-13), 28.3 (C-6), 26.6 (C-7), 24.5 (C-14), 23.8 (C-2), 20.3 (C-15), 20.2 (C-8), 19.6 (C-11), 16.3 (C-12).

1*β*, 6*α*-Dihydroxy-4(15)-eudesmene (3) Colorless oil, ESI-MS *m/z* 237 $[M-H]^-$. ^{1}H-NMR (600 MHz, $CDCl_3$) δ (ppm): 5.02 (1H, d, *J* = 1.2 Hz, H-15a), 4.74 (1H, d, *J* = 1.2 Hz, H-15b), 3.71 (1H, t, *J* = 10.2 Hz, H-6), 3.42 (1H, dd, *J* = 4.8, 11.4 Hz, H-1), 2.33 (1H, ddd, *J* = 2.4, 4.8, 13.2 Hz, H-3a), 2.24 (1H, td, *J* = 2.4, 7.2 Hz, H-11), 2.07 (1H, td, *J* = 2.4, 4.8 Hz, H-3b), 1.92 (1H, td, *J* = 3.0, 12.0 Hz, H-9a), 1.87 (1H, m, H-2a), 1.74 (1H, d, *J* = 10.2 Hz, H-5), 1.55 (2H, m, H-2b, H-8a), 1.29 (1H, m, H-7), 1.24 (1H, m, H-8b), 1.18 (1H, m, H-9b), 0.96 (3H, d, *J* = 7.2 Hz, H-12), 0.87 (3H, d, *J* = 7.2 Hz, H-13), 0.71 (3H, s, H-14). ^{13}C NMR (150 MHz, $CDCl_3$) δ (ppm): 146.2 (C-4), 107.8 (C-15), 79.0 (C-1), 67.0 (C-6), 55.9 (C-5), 49.3 (C-7), 41.7 (C-10), 36.3 (C-9), 35.1 (C-3), 31.9 (C-2), 26.0 (C-11), 21.1 (C-12), 18.2 (C-8), 16.2 (C-13), 11.6 (C-14).

1*β*H,5*β*H-aromandendrane-4*α*,10*β*-diol (4) Colorless oil, ESI-MS *m/z* 237 $[M-H]^-$. ^{1}H-NMR (600 MHz, $CDCl_3$) δ (ppm): 2.48 (1H, m, H-1), 1.86 (1H, m, H-2), 1.34 (3H, s, H-15), 1.20 (3H, s, H-14), 1.04 and 1.03 (each 3H, s, H-12 and H-13), 0.88 (1H, m, H-8a), 0.62 (1H, m, H-7). ^{13}C NMR (150 MHz, $CDCl_3$) δ (ppm): 82.1 (C-4), 74.3 (C-10), 54.1 (C-1), 47.8 (C-5), 38.0 (C-9), 37.5 (C-3),

32.1 (C-15), 28.8 (C-7), 28.6 (C-13), 25.6 (C-14), 25.4 (C-6), 25.2 (C-2), 18.8 (C-8), 18.7 (C-11), 16.2 (C-12).

Acetyl oleanolic acid (5): White amorphous powder; ESI-MS *m/z* 499 $[M+H]^+$.

^{1}H-NMR (600 MHz, $CDCl_3$) δ (ppm): 5.25 (1H, t, J = 3.6 Hz, H-12), 4.49 (1H, m, H-3α), 2.04 (3H, s, CH_3-31), 1.06 (3H, s, CH_3-27), 0.95 (3H, s, CH_3-26), 0.94 (3H, d, J = 7.2 Hz, CH_3-29), 0.86 (3H, s, CH_3-23), 0.85 (3H, d, J = 6.6 Hz, CH_3-30), 0.84 (3H, s, CH_3-24), 0.78 (3H, s, CH_3-25). ^{13}C NMR (150 MHz, $CDCl_3$) δ (ppm): 182.1 (C-28), 171.0 (C-31), 138.1 (C-13), 125.7 (C-12), 80.9 (C-3), 55.3 (C-5), 52.7 (C-18), 47.9 (C-17), 47.5 (C-9), 42.0 (C-14), 39.5 (C-8), 39.0 (C-20), 38.8 (C-19), 38.3 (C-1), 37.7 (C-4), 37.7 (C-22), 37.3 (C-10), 32.9 (C-7), 30.6 (C-21), 28.1 (C-23), 28,0 (C-15), 24.1 (C-16), 23.6 (C-27), 23.5 (C-11), 23.3 (C-2), 21.3 (C-30), 21.4 (C-32), 18.2 (C-6), 17.1 (C-29), 17.0 (C-26), 15.5 (C-25).

3*α*-Hydroxytirucalla-8,24-dien-21-oic acid (6) White amorphous powder; ESI-MS *m/z* 457 $[M+H]^+$. ^{1}H-NMR (600 MHz, $CDCl_3$) δ (ppm): 5,09 (1H, t, J = 7,2 Hz, H-24), 3,45 (1H, t, J = 2,4 Hz, H-3), 2,29 (1H, m, H-20), 1,67 (3H, s, H-27), 1.58 (3H, s, H-26), 0,96 (3H, s, H-28), 0.88 (3H, s, H-30), 0.86 (3H, s, H-29), 0.83 (3H, s, H-18). ^{13}C-NMR (150 MHz, $CDCl_3$): 180.1 (C-21), 134.3 (C-9), 132.9 (C-8), 132.1 (C-25), 123.6 (C-24), 76.1 (C-3), 49.6 (C-14), 47.3 (C-20), 46.9 (C-17), 44.8 (C-5), 43.8 (C-13), 37.6 (C-4), 37.2 (C-10), 32.5 (C-1), 29.8 (C-22), 29.3 (C-15), 28.9 (C-12), 27.1 (C-16), 26.9 (C-7), 25.9 (C-2), 25.7 (C-23), 25.6 (C-27), 24.5 (C-30), 22.2 (C-29), 21.5 (C-11), 19.8 (C-19), 18.7 (C-6), 17.6 (C-26), 15.8 (C-18).

Ursolic acid (7): White amorphous powder, ESI-MS *m/z* 457 $[M+H]^+$. ^{1}H-NMR (500 MHz, $CDCl_3$+ CD_3OD) δ (ppm): 5.16 (1H, t, J = 7.5 Hz, H-12), 3.12 (1H, m, H-3), 1.01 (3H, s, H-27), 0.90 (3H, s, H-23), 0.86 (3H, d, J = 6.5 Hz, H-29), 0.85 (3H, s, H-24), 0.74 (3H, s, H-26), 0.70 (3H, s, H-25), 0.80 (3H, d, J = 6.5 Hz, H-30). ^{13}C-NMR (125 MHz, $CDCl_3$+ CD_3OD): 180.5 (C-28), 138.0 (C-13), 125.4 (C-12), 78.8 (C-3), 55.1 (C-5), 52.7 (C-18), 47.7 (C-17), 47.5 (C-9), 41.9 (C-14), 39.3 (C-8), 39.0 (C-4), 38.8 (C-19), 38.6 (C-20), 38.5 (C-1), 36.8 (C-10), 36.7 (C-22), 32.9 (C-7), 30.6 (C-21), 27.9 (C-23), 27.9 (C-2), 26.7 (C-15), 24.1 (C-16), 23.4 (C-27), 23.1 (C-11), 21.0 (C-30), 18.2 (C-6), 16.9 (C-29), 16.8 (C-26), 15.47 (C-25), 15.2 (C-24).

Cochinchinone A (8): yellow solid. ESI-MS $[M+H]^+$ 449. ^{1}H-NMR (600 MHz, $CDCl_3$) δ (ppm): 13,12 (1H, s, 1-OH), 7,50 (1H, d, J = 3,0 Hz, H-8), 7,36 (1H, d, J = 8,4 Hz, H-5), 7,25 (1H, dd, J = 3,0; 8.4 Hz, H-6), 6,45 (1H, s, OH), 5,49 (1H, s, OH), 5,28 (2H, m, H-2', H-2''), 5,05 (1H, t, J = 7,2 Hz, H-6''), 3.56 (2H, d, J = 7,2 Hz, H-1''), 3,46 (2H, d, J = 6,6 Hz, H-1'), 2,09 (2H, m, H-5''), 2,05 (2H, m, H-4''), 1,88 (3H, s, H-9''), 1.85 (3H, s, H-4'), 1,76 (3H, s, H-5'), 1,63 (3H, s, H-8''), 1,57 (3H, s, H-10''). ^{13}C-NMR (150 MHz, $CDCl_3$): 180.1 (C-21), 134.3 (C-9), 132.9 (C-8), 132.1 (C-25), 123.6 (C-24), 76.1 (C-3), 49.6 (C-14), 47.3 (C-20), 46.9 (C-17), 44.8 (C-5), 43.8 (C-13), 37.6 (C-4),

37.2 (C-10), 32.5 (C-1), 29.8 (C-22), 29.3 (C-15), 28.9 (C-12), 27.1 (C-16), 26.9 (C-7), 25.9 (C-2), 25.7 (C-23), 25.6 (C-27), 24.5 (C-30), 22.2 (C-29), 21.5 (C-11), 19.8 (C-19), 18.7 (C-6), 17.6 (C-26), 15.8 (C-18).

***α*-mangostin (9):** yellow solid. ESI-MS $[M+H]^+$ 411. **^{1}H-NMR** (500 MHz, CD_3OD) δ (ppm): 6.70 (1H, s, H-5); 6.25 (1H, s, H-4); 5.25 (2H, m, H-2', H-2''); 4.09 (2H, d, *J* = 6.5Hz, H-1''); 3.77 (3H, s, 7-OMe), 3.30 (2H, d, *J* = 7.5 Hz, H-1'); 1.84 (3H, s, H-4''); 1.79 (3H, s, H-5''); 1.69 (3H, s, H-4'); 1.67 (3H, s, H-5'). **^{13}C-NMR** (125 MHz, CD_3OD) δ (ppm): 183.1 (C-9); 163.6 (C-1); 161.5 (C-3); 157.9 (C-4a); 156.7 (C-4b); 156.1 (C-6); 144.8 (C-7); 138.4 (C-8); 131.8 (C-3''), 131.6 (C-3'), 125.1 (C-2''); 123.8 (C-2'); 112.1 (C-8a); 111.4 (C-2); 103.7 (C-9a); 102.7 (C-5), 93.1 (C-4), 61.3 (7-OMe), 27.1 (C-1''); 25.9 (C-4'); 25.9 (C-4''); 22.2 (C-1'); 18.3 (C-5''); 17.9 (C-5').

Table S1. Docking score of isolated compounds with *α*-glucosidase

No.	Compound	IC_{50} (μg/mL)	Docking score (kcal/mol)
1	**1**	>256	-6.6
2	**2**	>256	-7.0
3	**3**	>256	-7.0
4	**4**	>256	-7.0
5	**5**	8.44±0.23	-8.0
6	**6**	>256	-7.2
7	**7**	25.34 ± 0.54	-6.1
8	**8**	9.07±0.65	-9.3
9	**9**	1.90±0.10	-9.9
**10**	**Acarbose**	**134.56±3.02**	**-7.6**

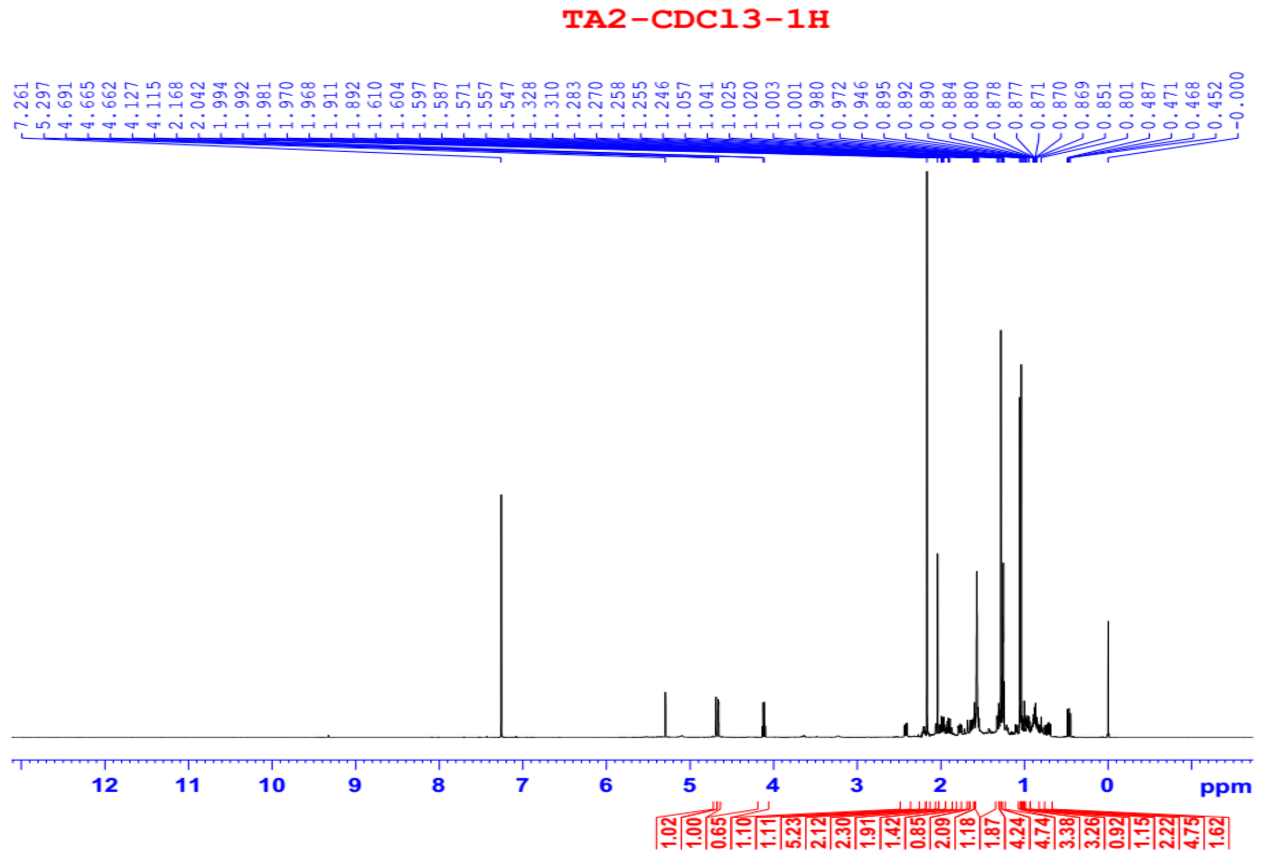

Figure S1. ^{1}H-NMR spectrum of compound **1**

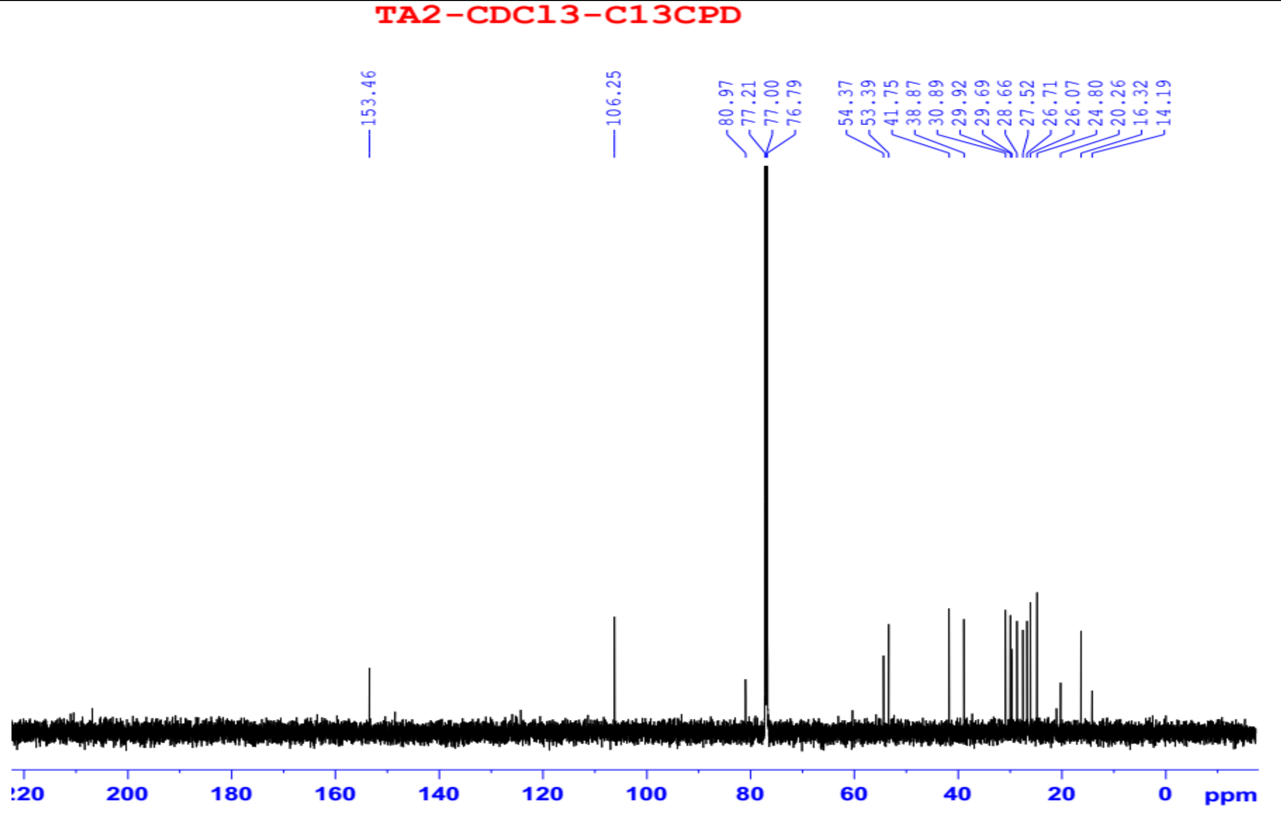

Figure S2. ^{13}C-NMR spectrum of compound **1**

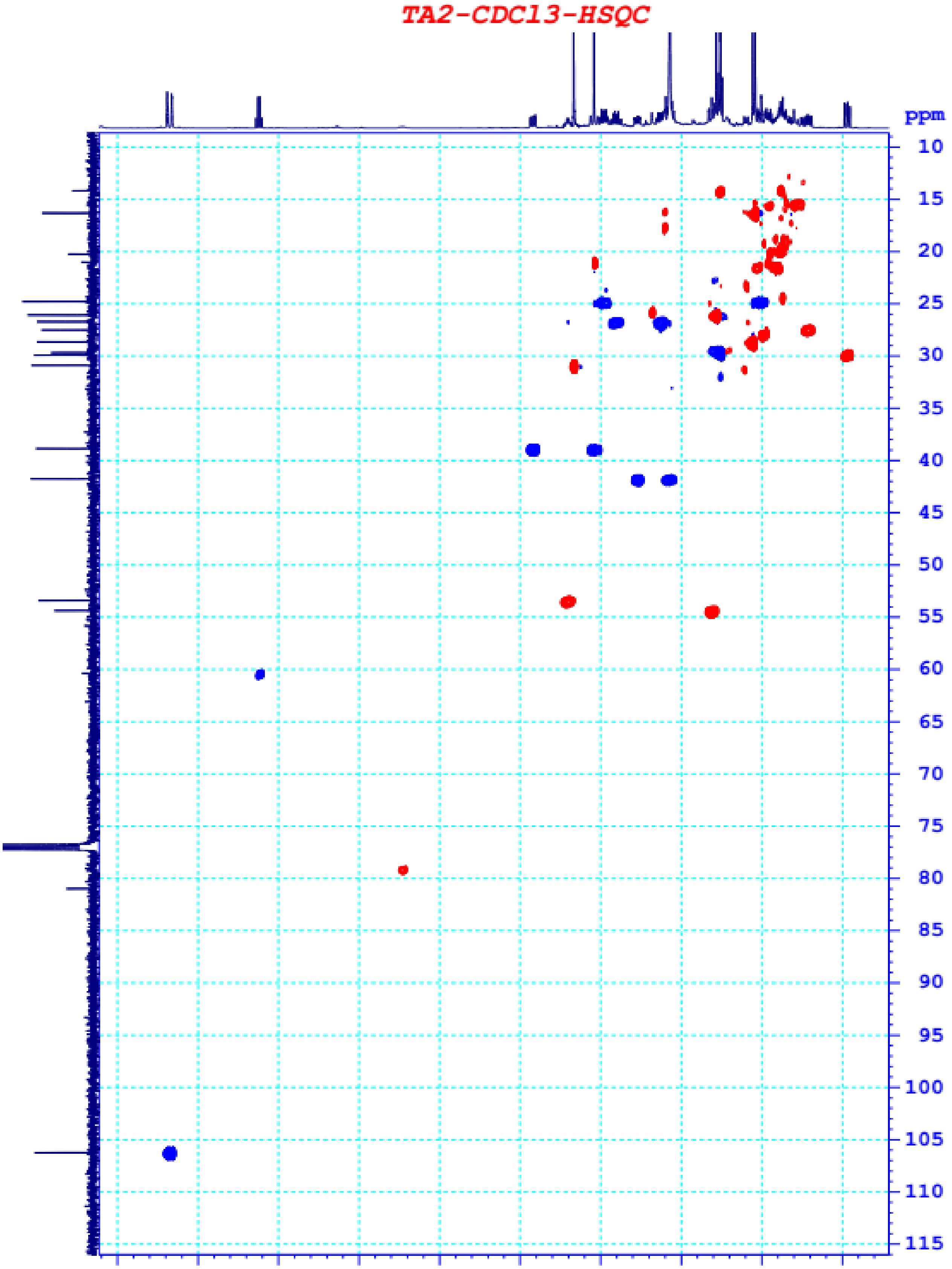

Figure S3. HSQC spectrum of compound **1**

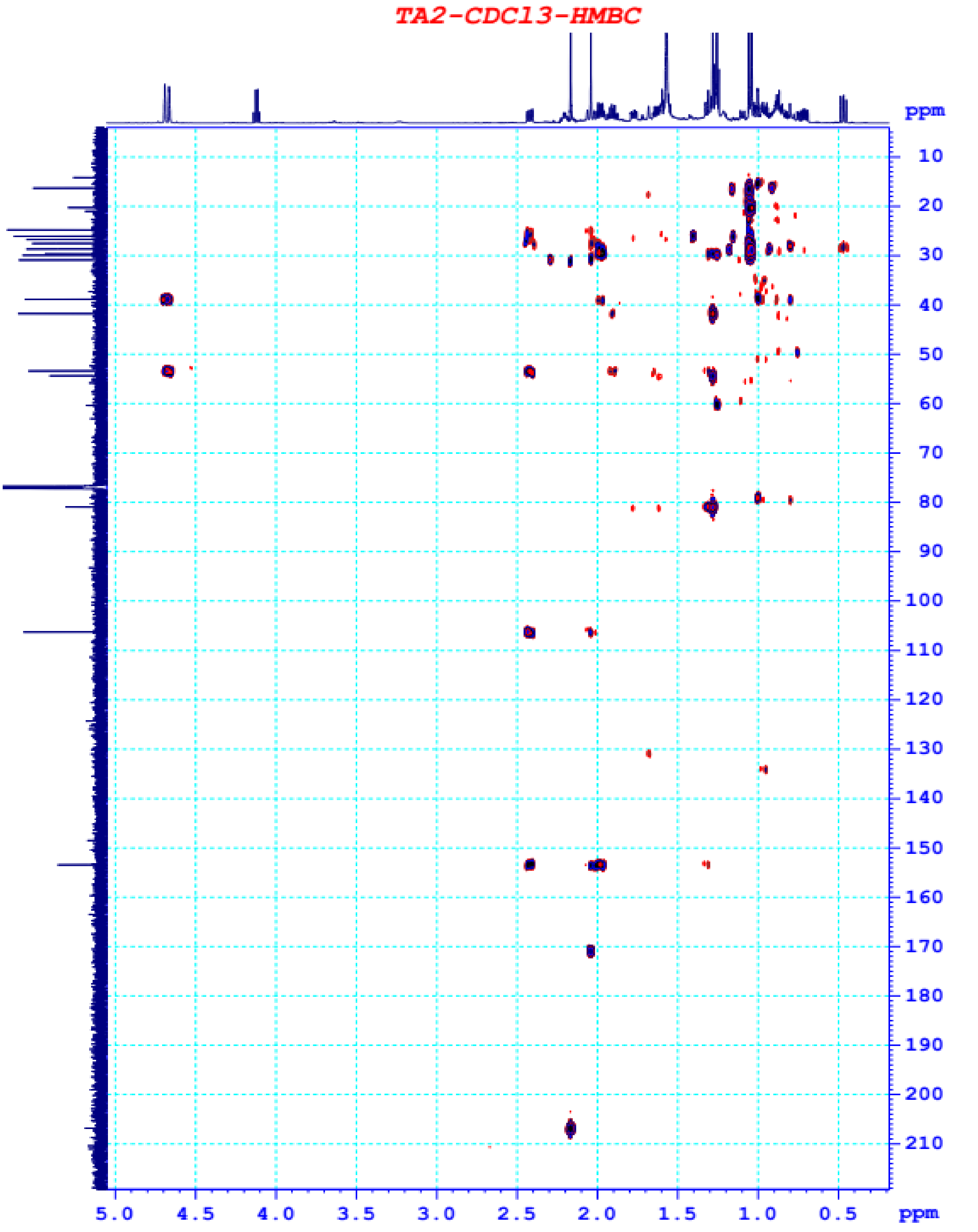

Figure S4. HMBC spectrum of compound **1**

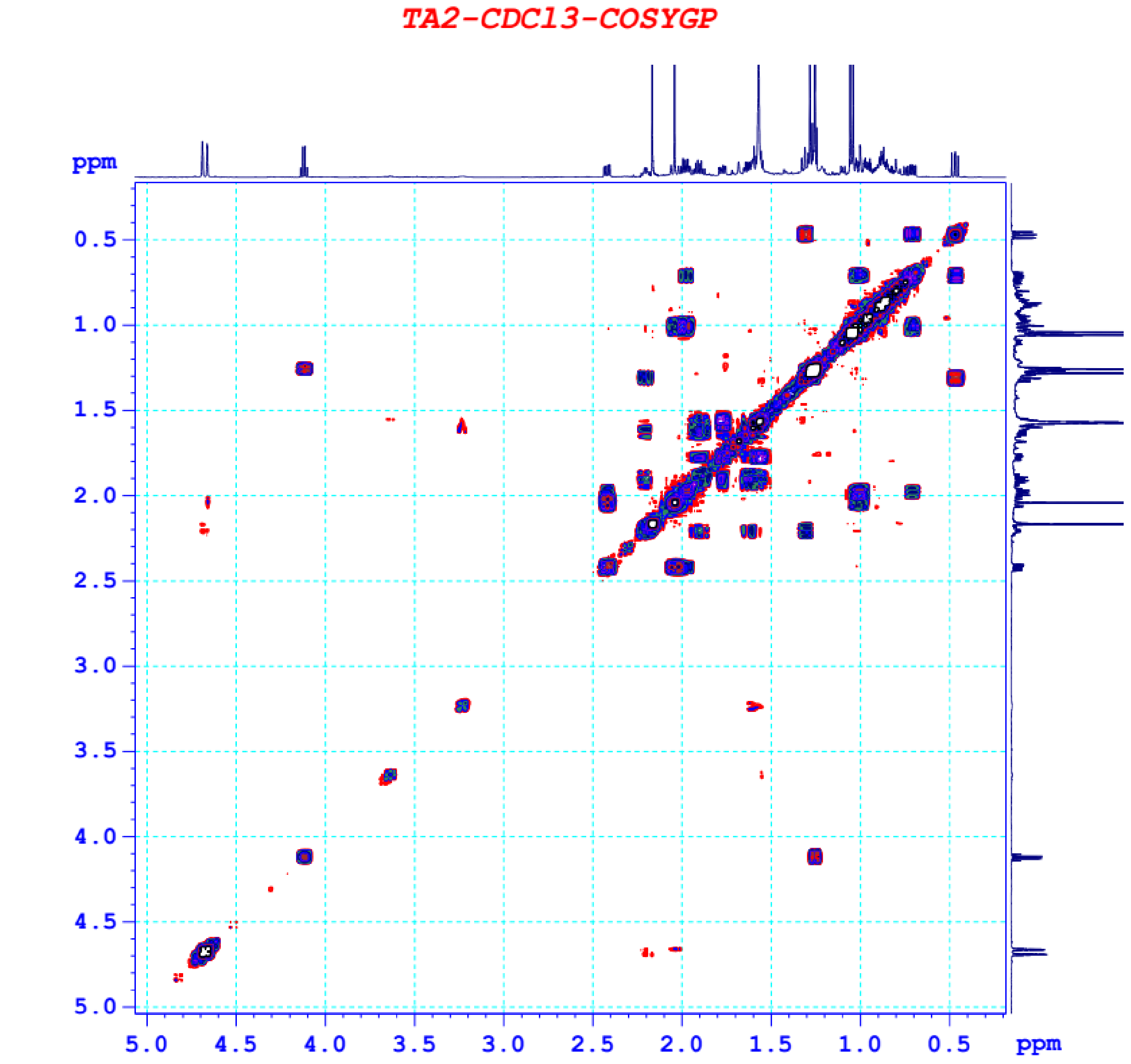

Figure S5. ^{1}H-^{1}H COSY spectrum of compound **1**

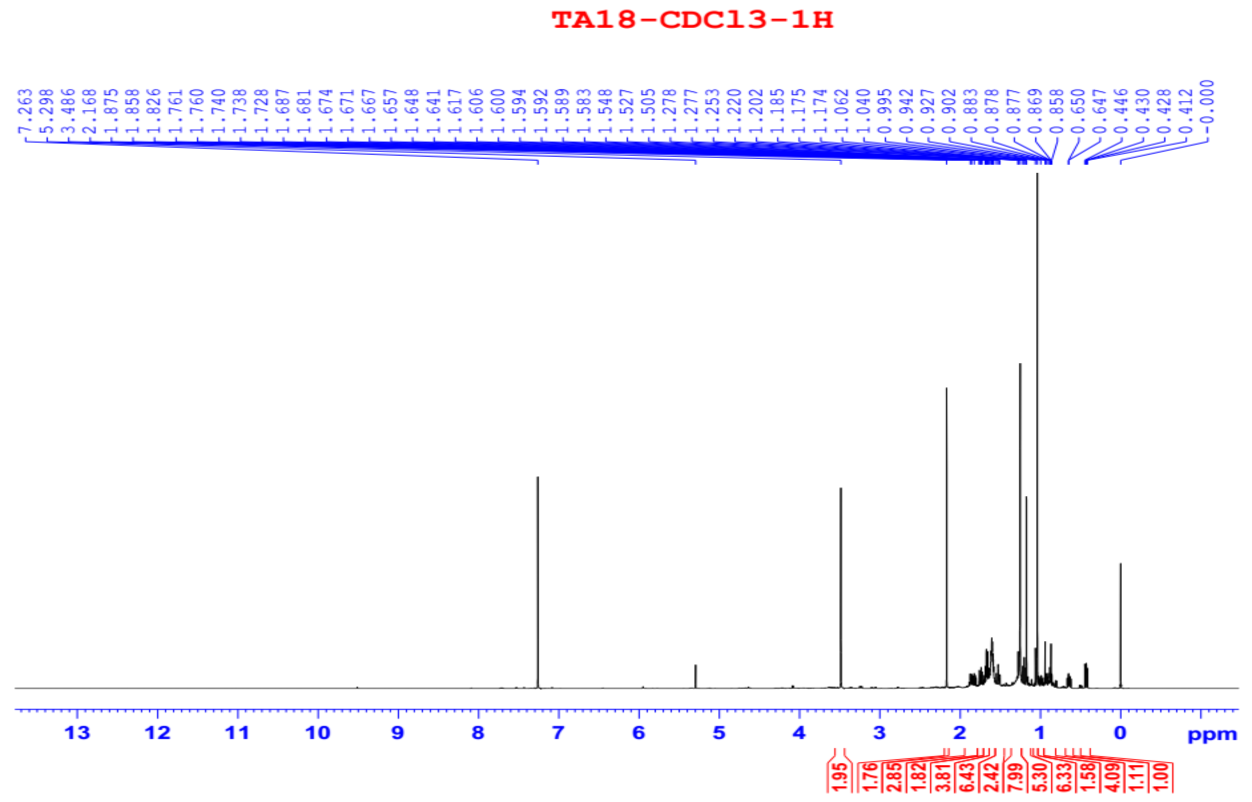

Figure S6. ^{1}H-NMR spectrum of compound **2**

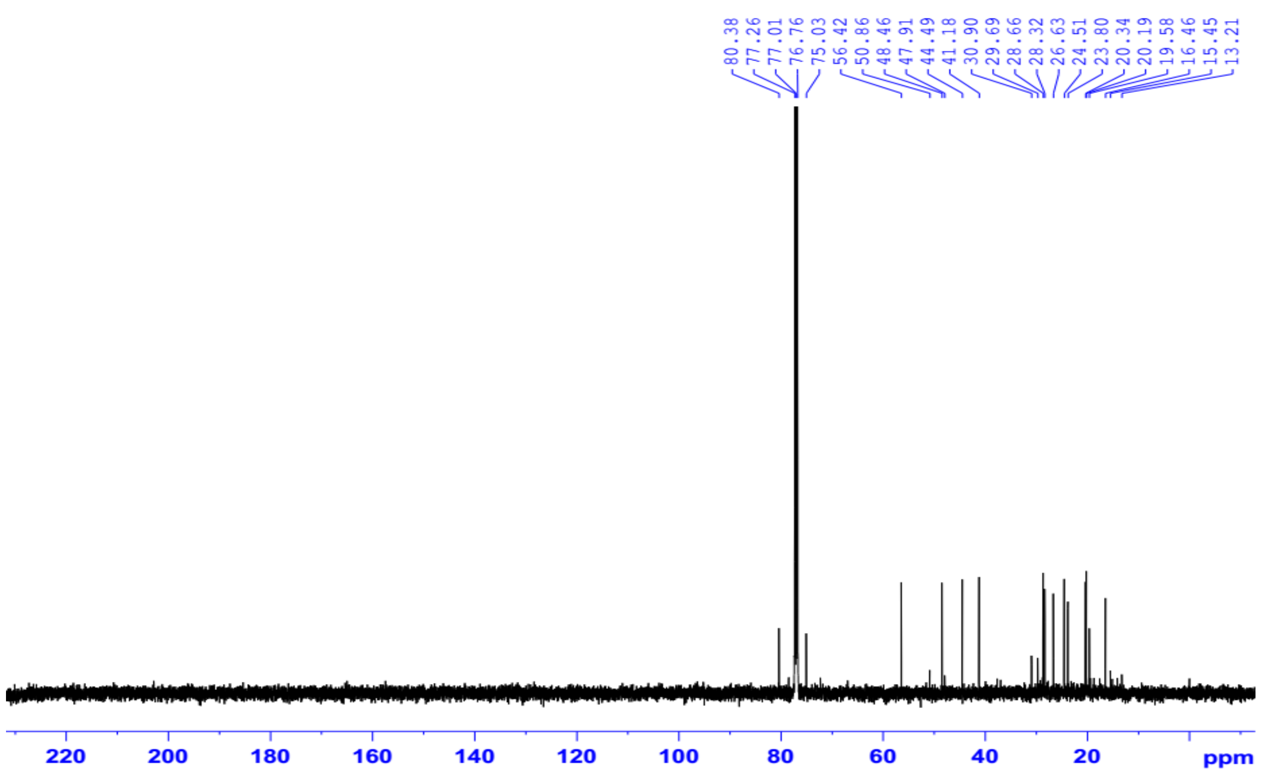

Figure S7. ^{13}C-NMR spectrum of compound **2**

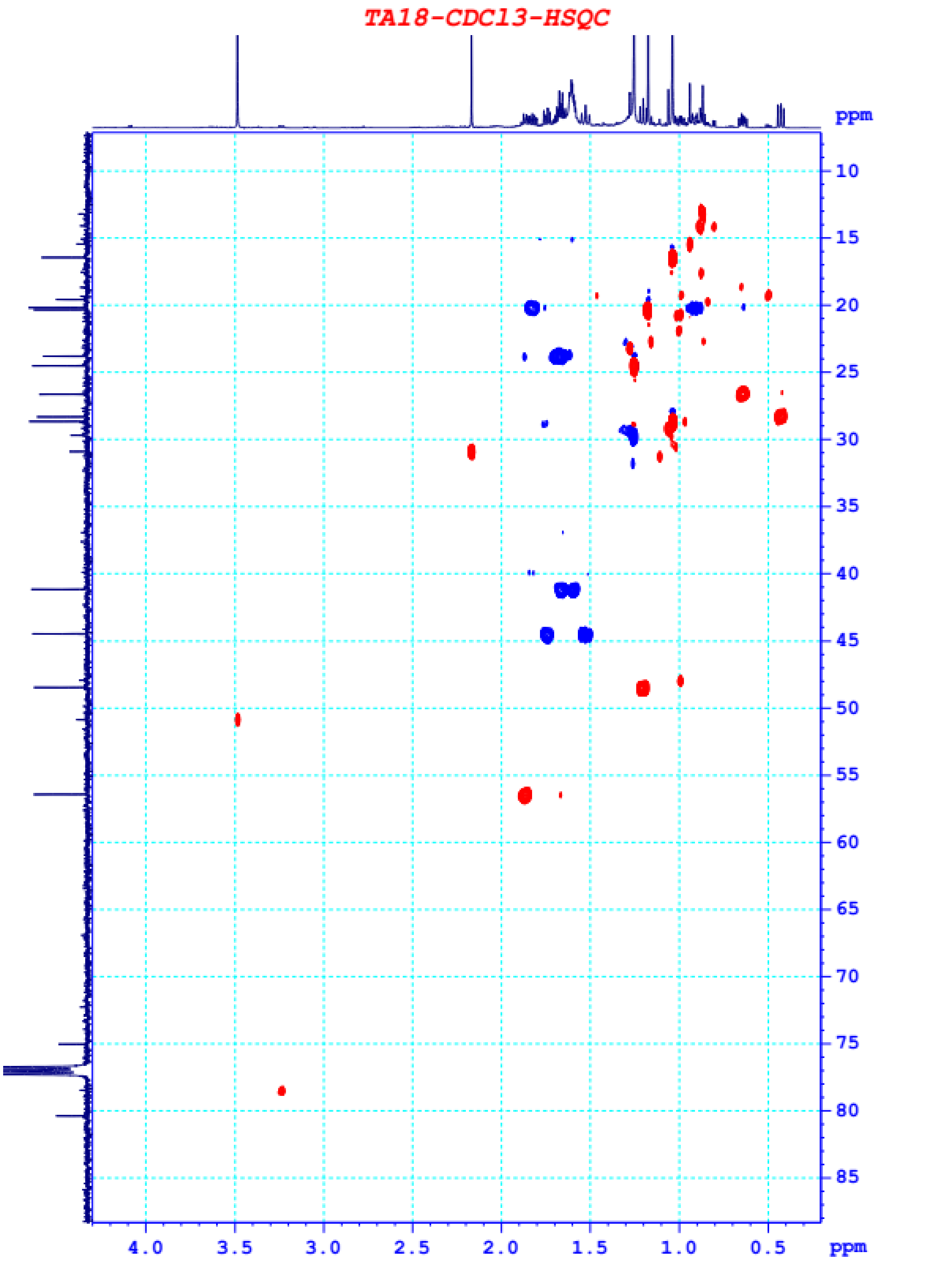

Figure S8. HSQC spectrum of compound **2**

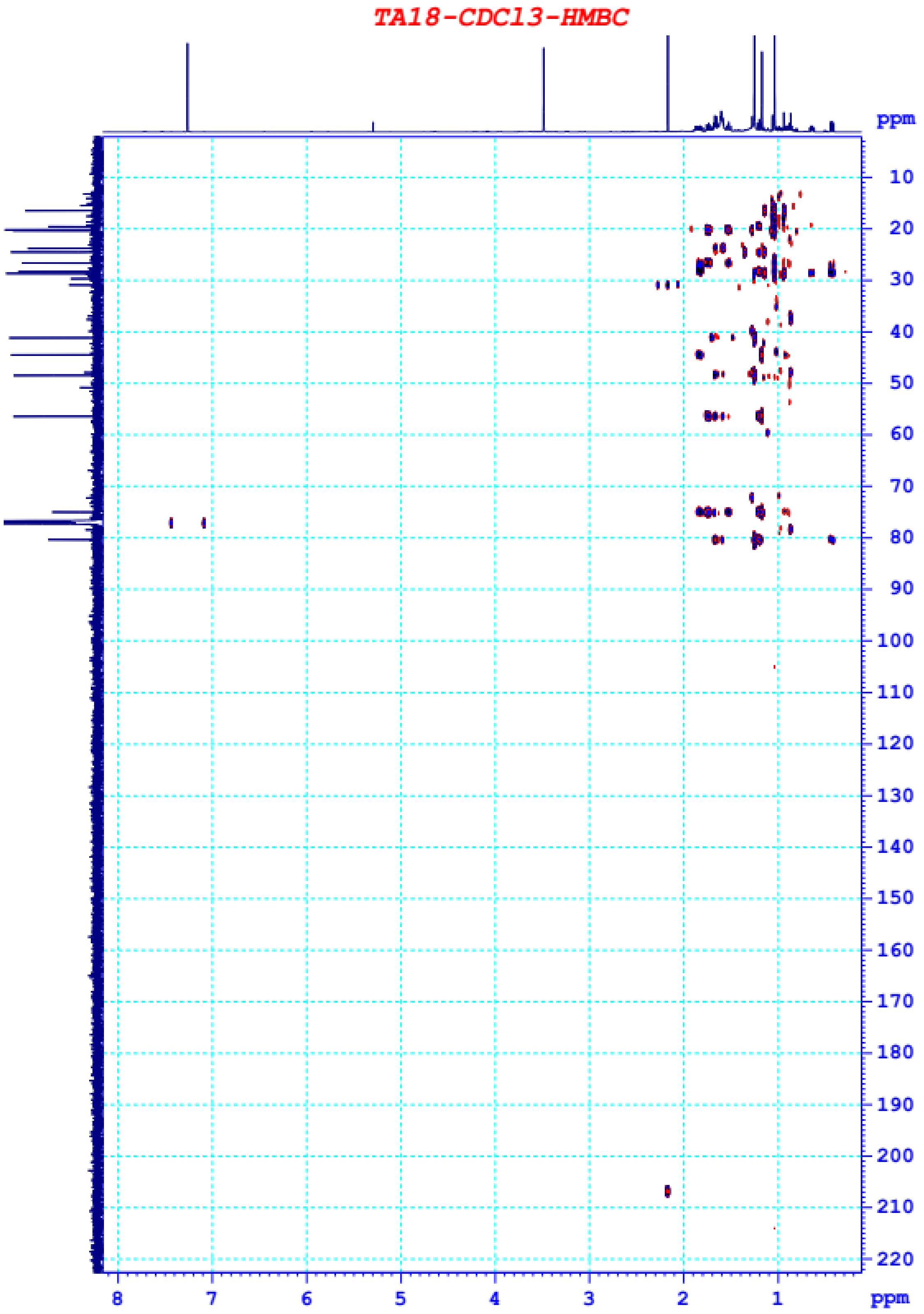

Figure S9. HMBC spectrum of compound **2**

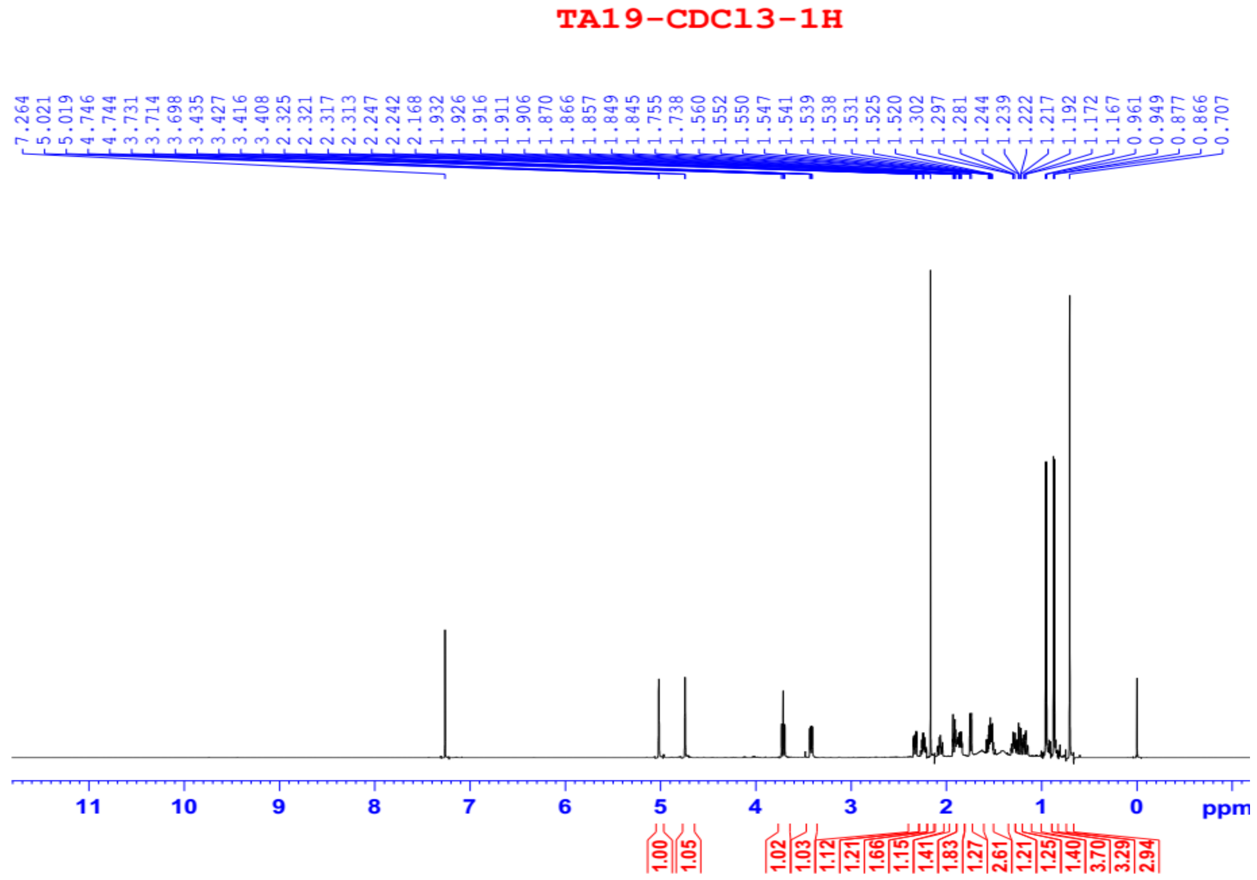

Figure S10. ^{1}H-NMR spectrum of compound **3**

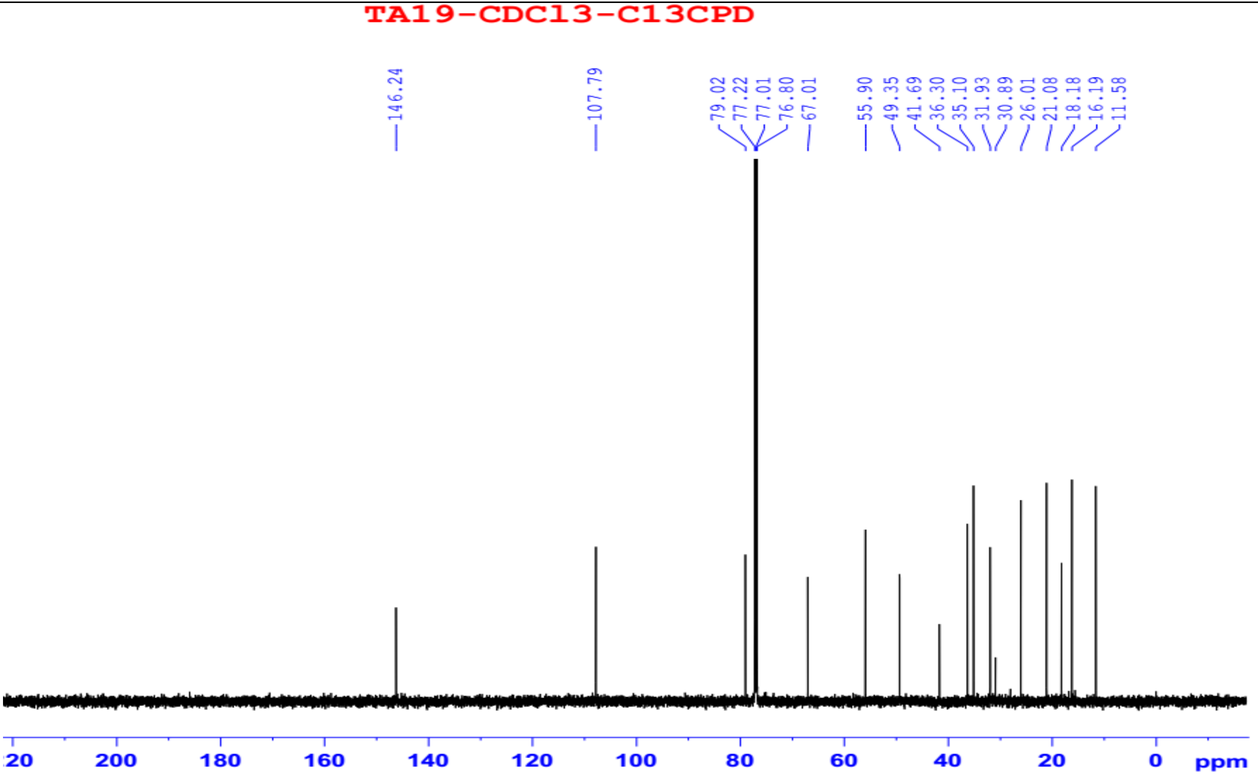

Figure S11. ^{13}C-NMR spectrum of compound **3**

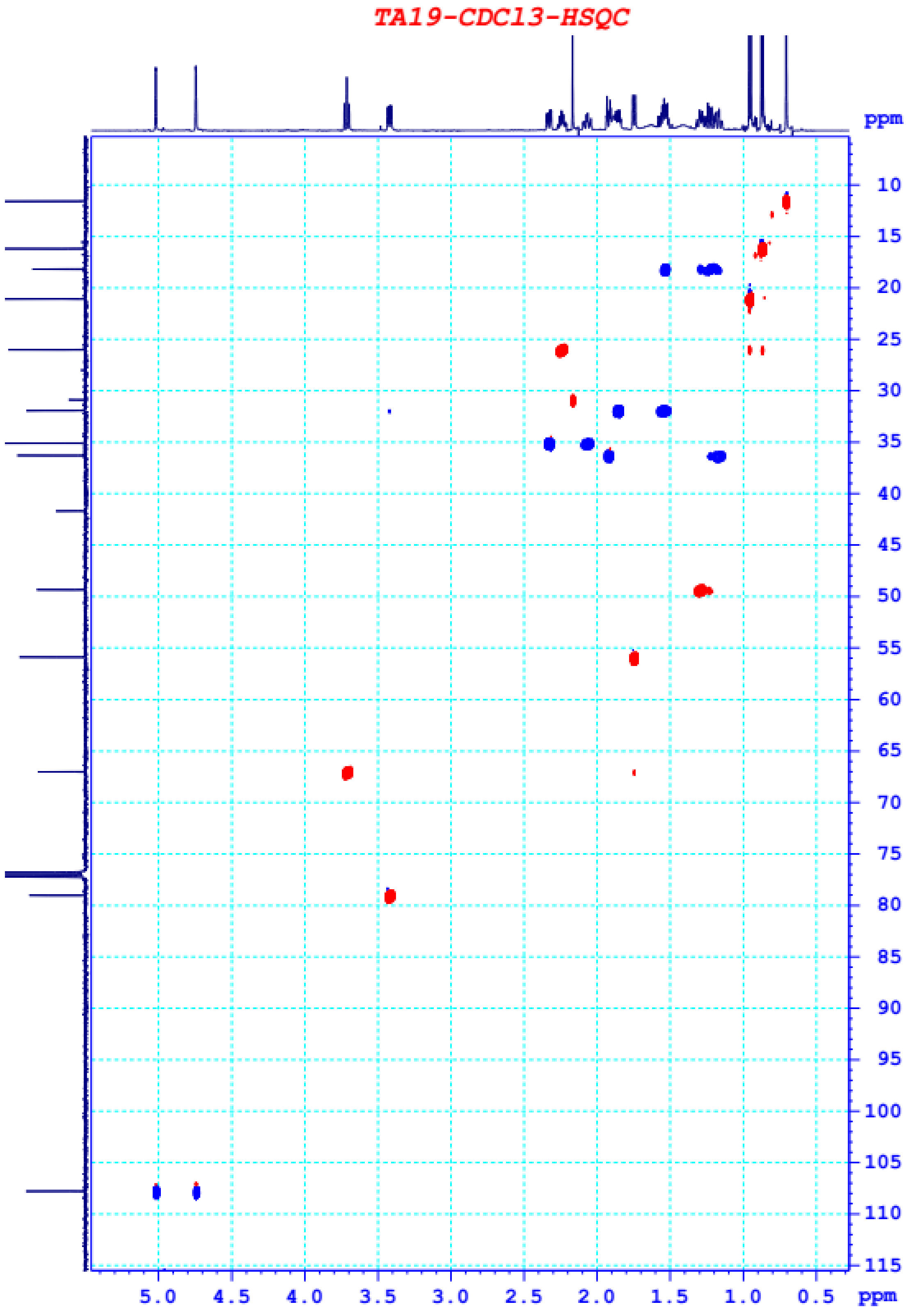

Figure S12. HSQC spectrum of compound **3**

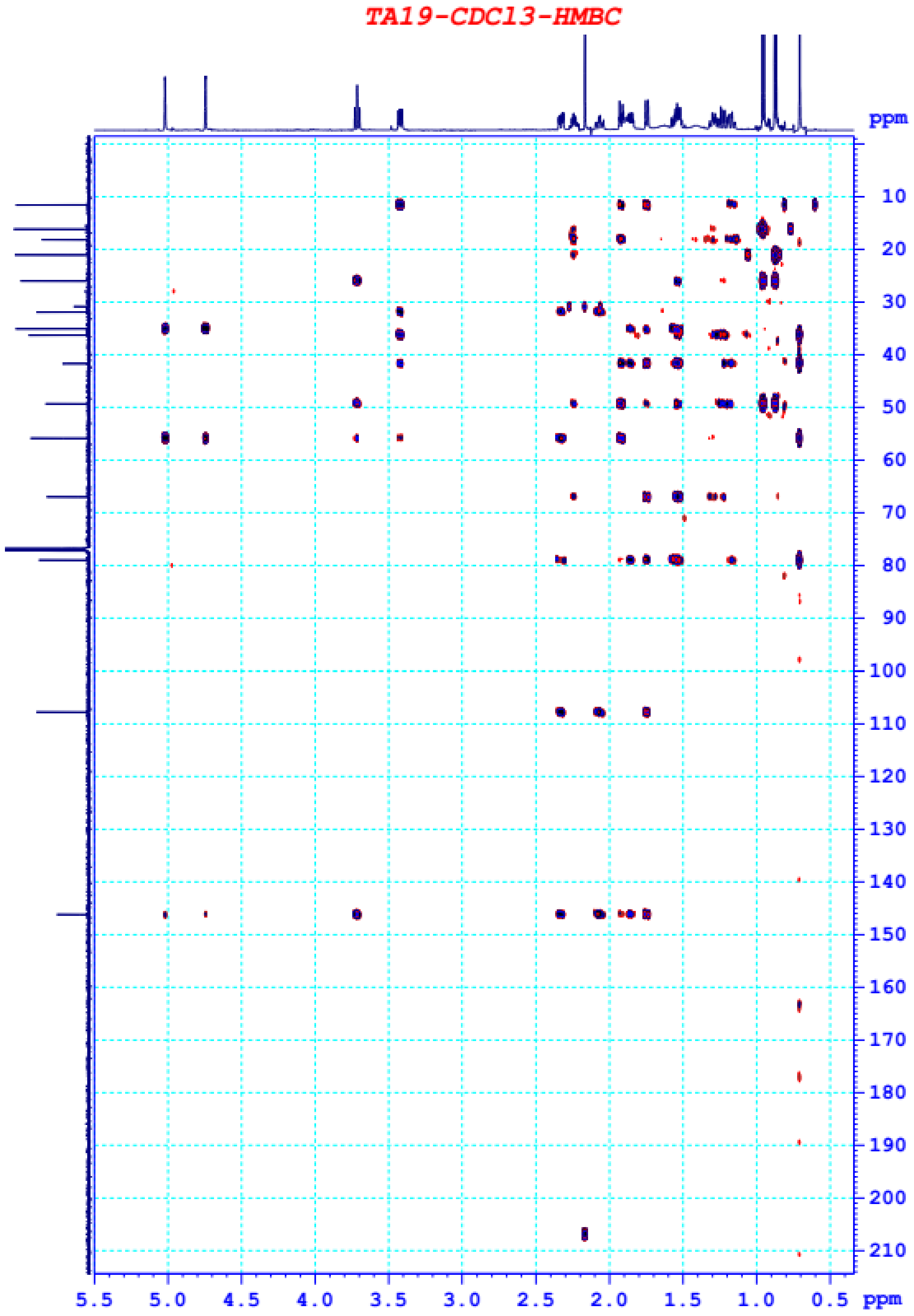

Figure S13. HMBC spectrum of compound **2**

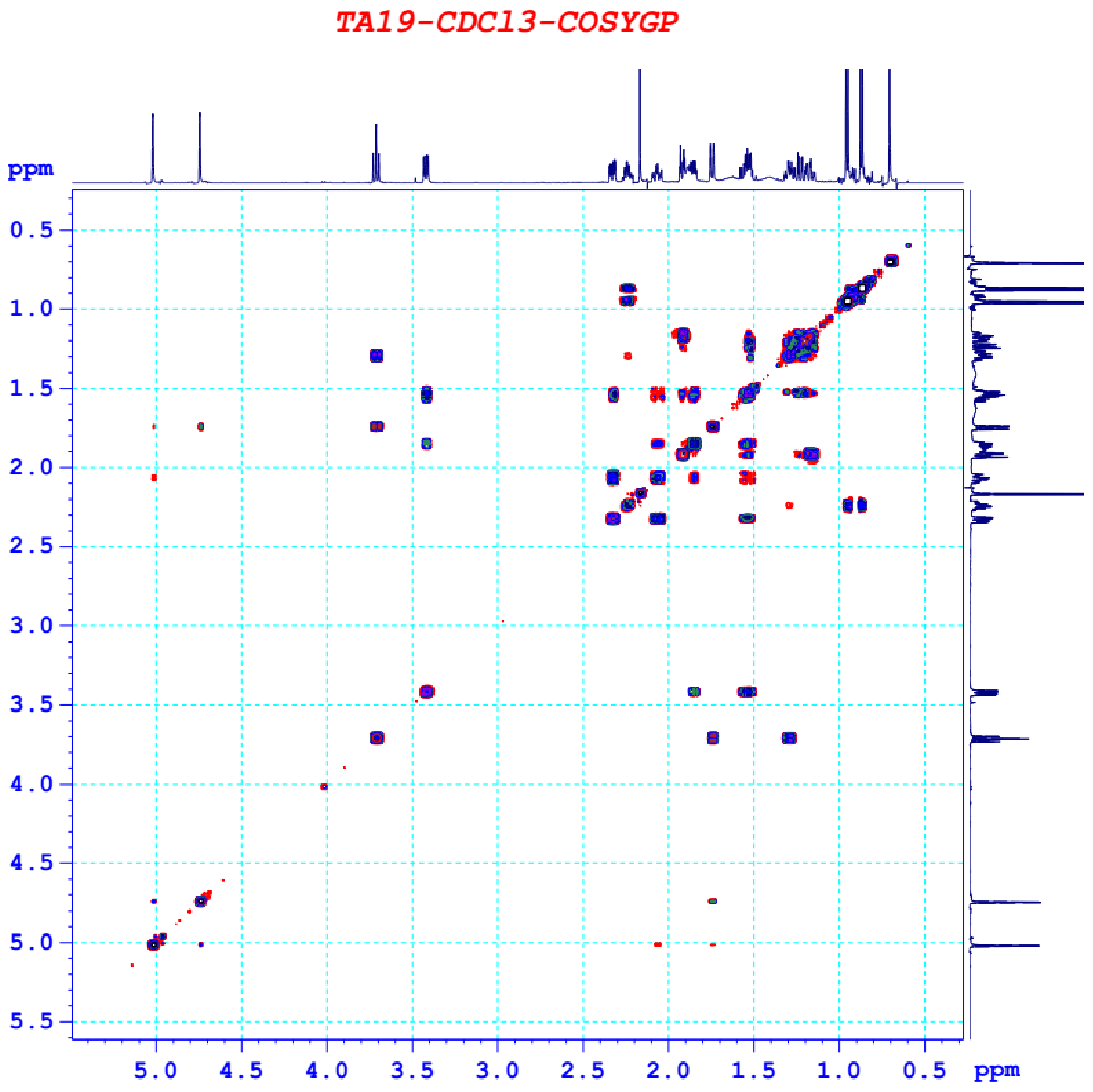

Figure S14. ^{1}H-^{1}H COSY spectrum of compound **3**

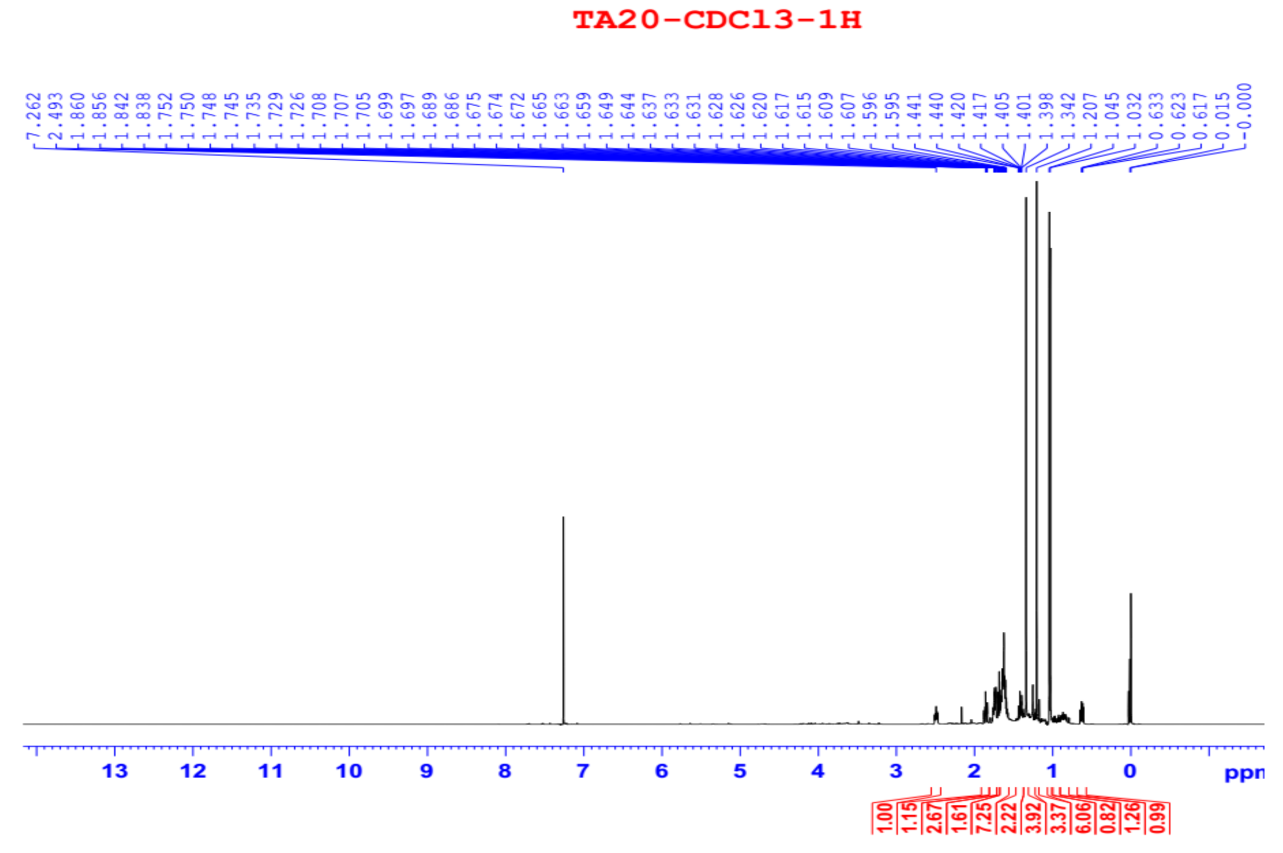

Figure S15. ^{1}H-NMR spectrum of compound **4**

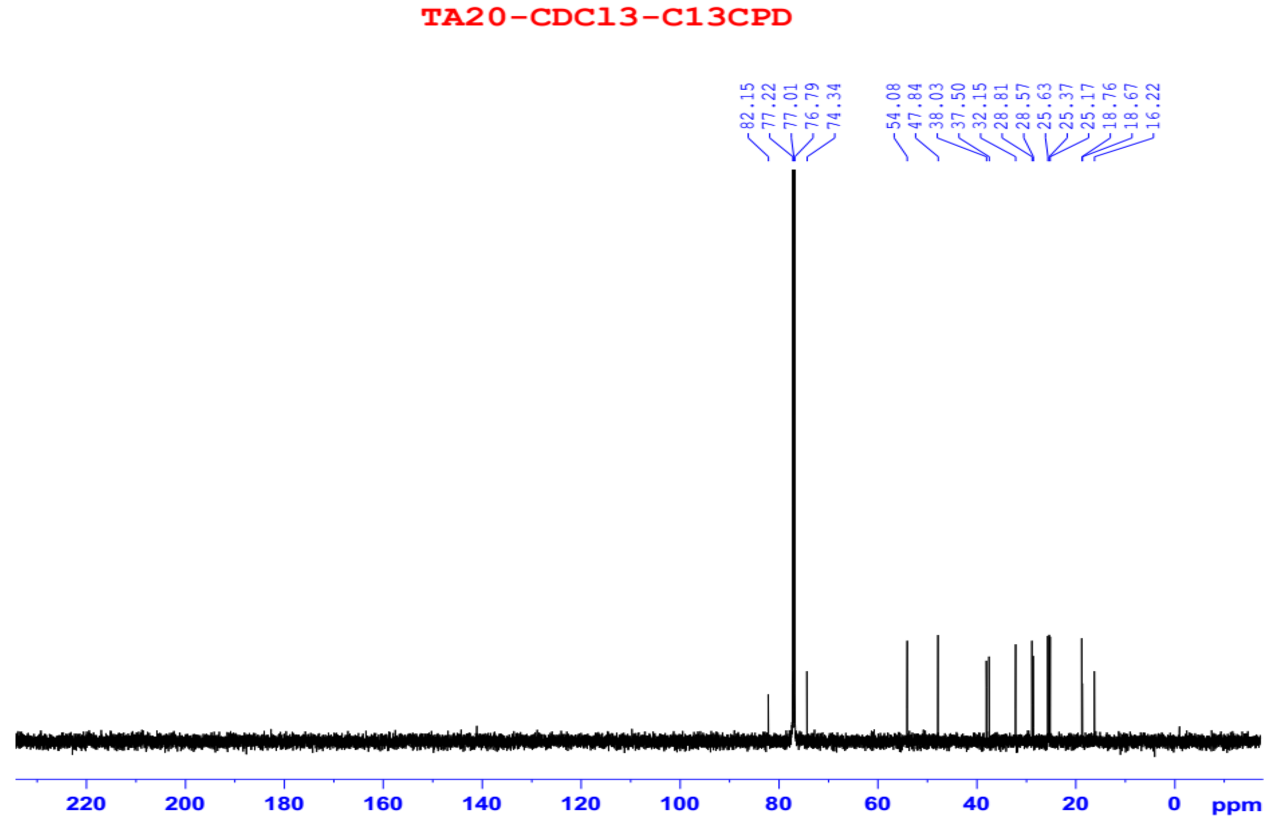

Figure S16. ^{13}C-NMR spectrum of compound **4**

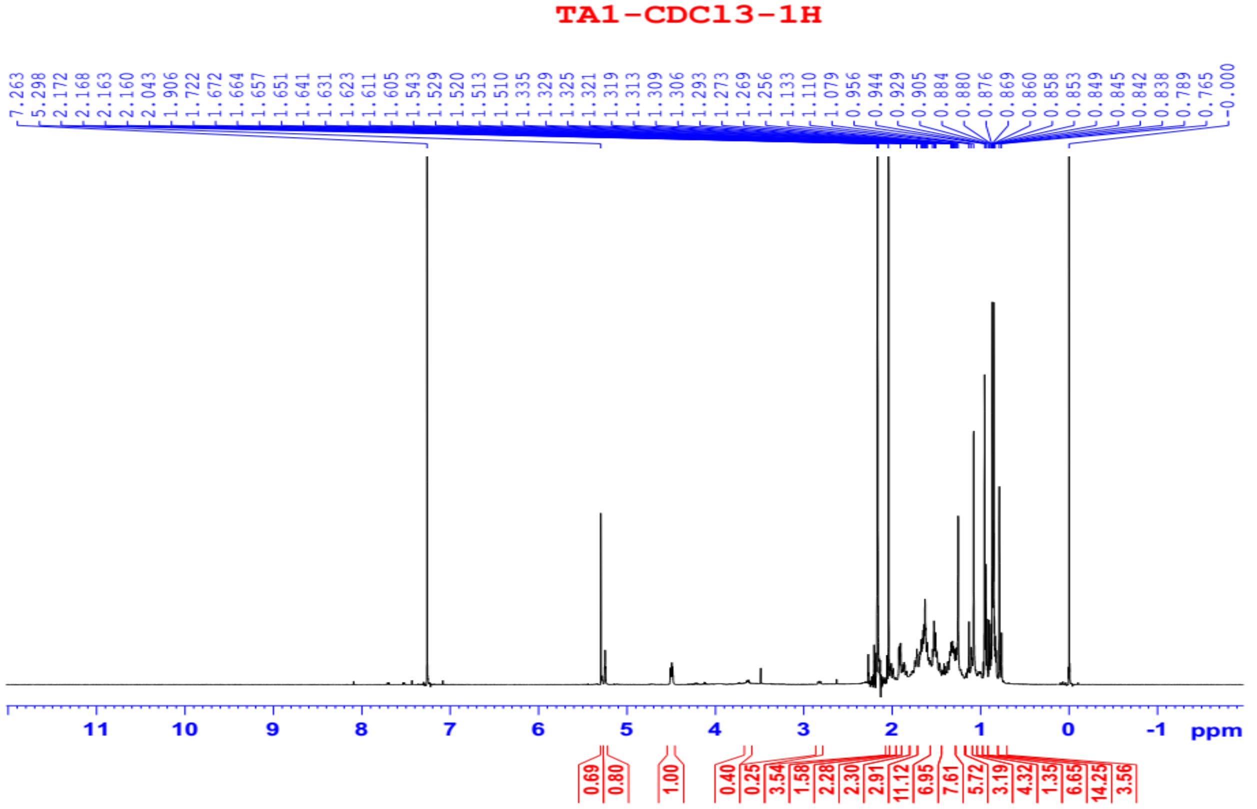

Figure S17. ^{1}H-NMR spectrum of compound **5**

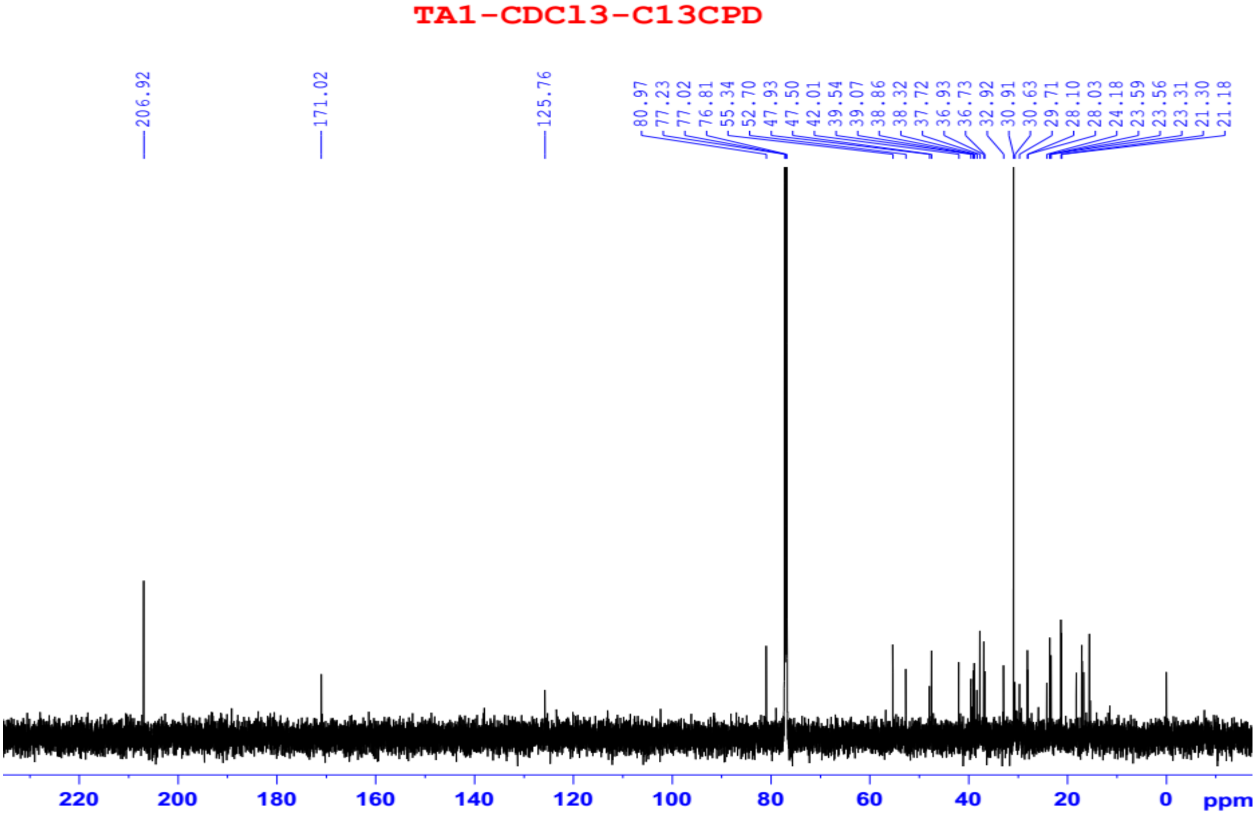

Figure S18. ^{13}C-NMR spectrum of compound **5**

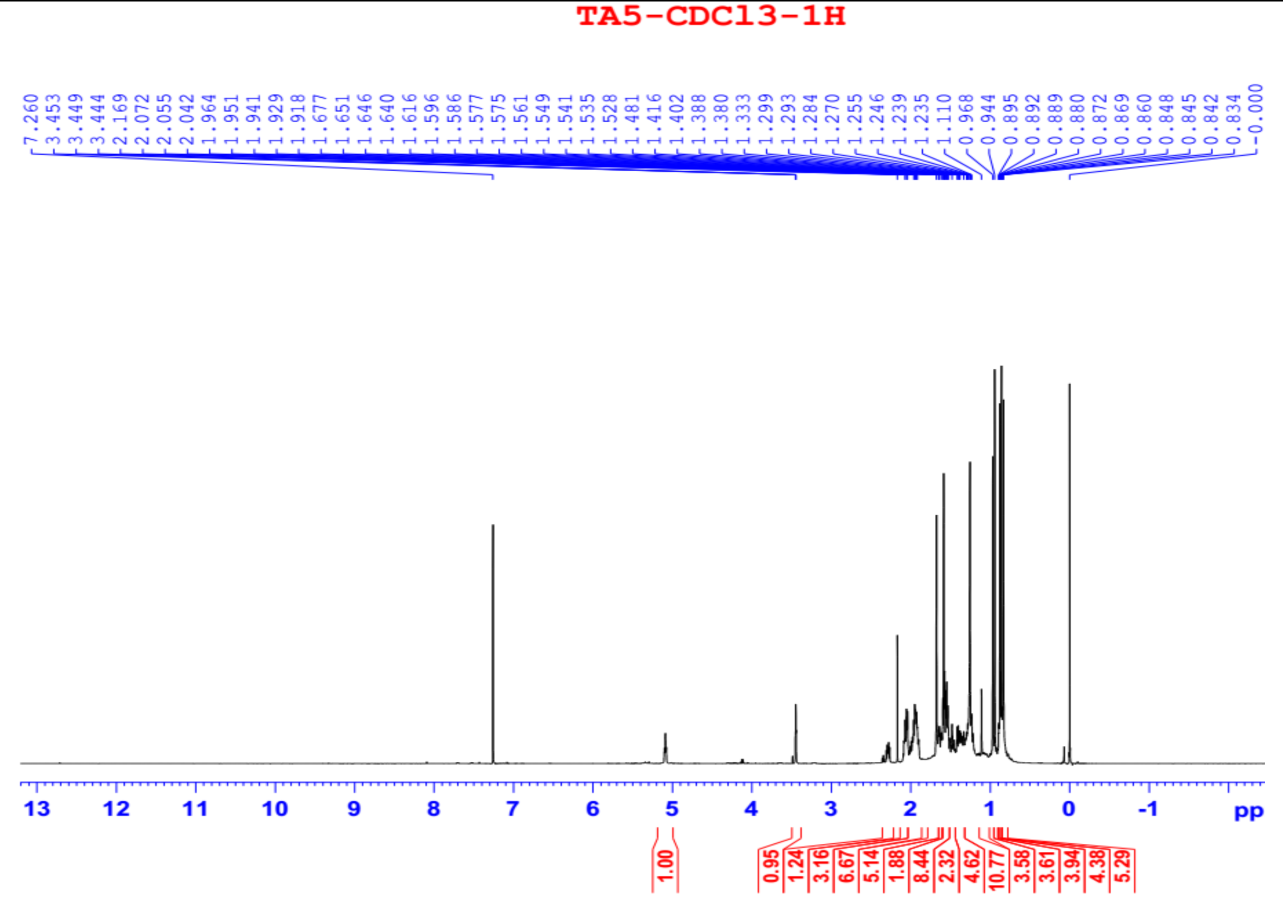

Figure S19. ^{1}H-NMR spectrum of compound **6**

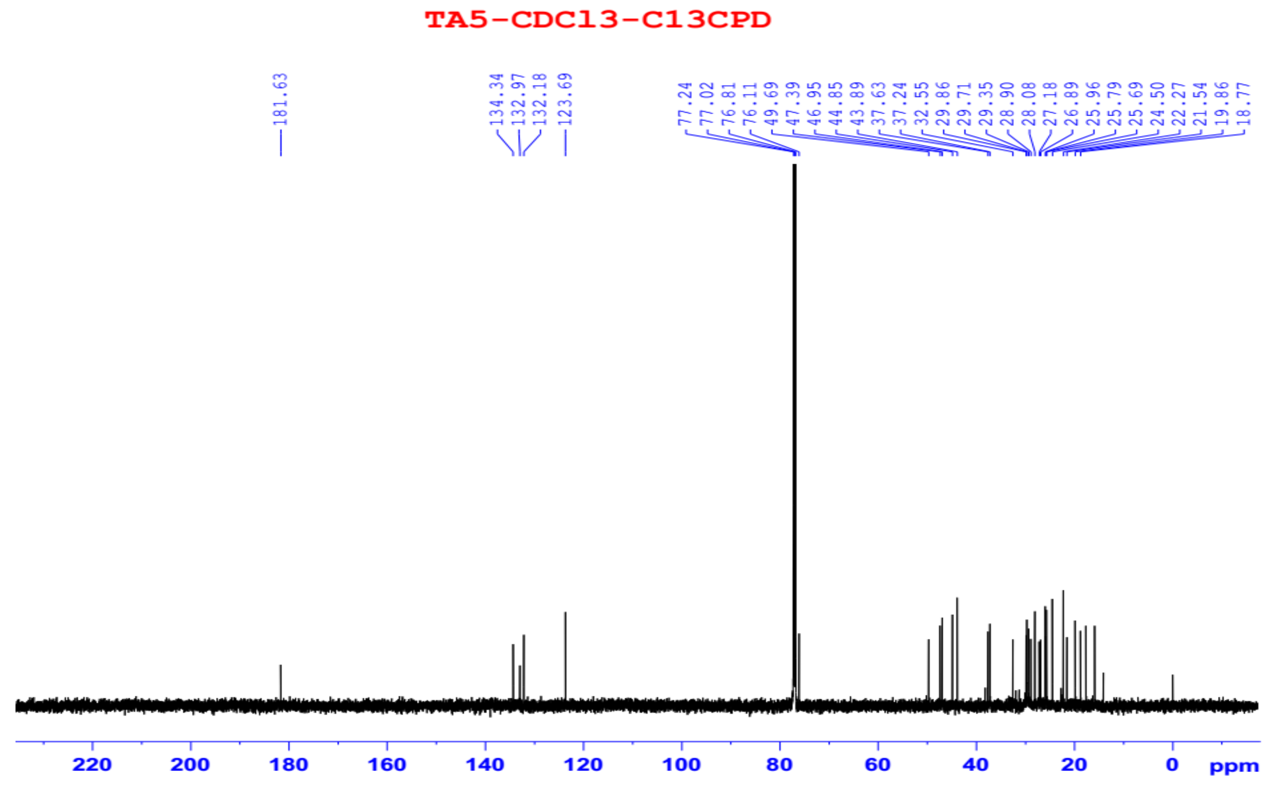

Figure S20. ^{13}C-NMR spectrum of compound **6**

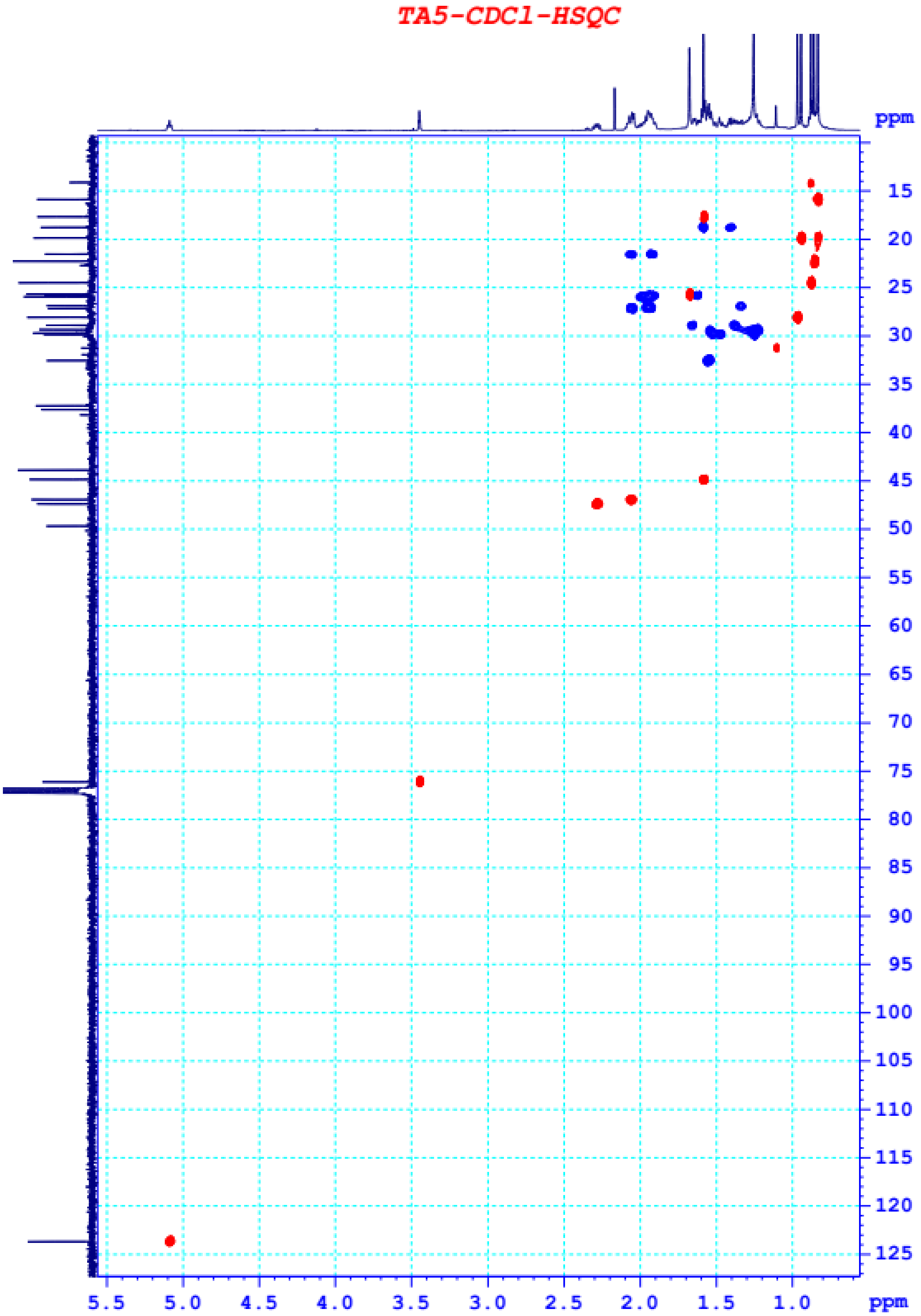

Figure S21. HSQC spectrum of compound **6**

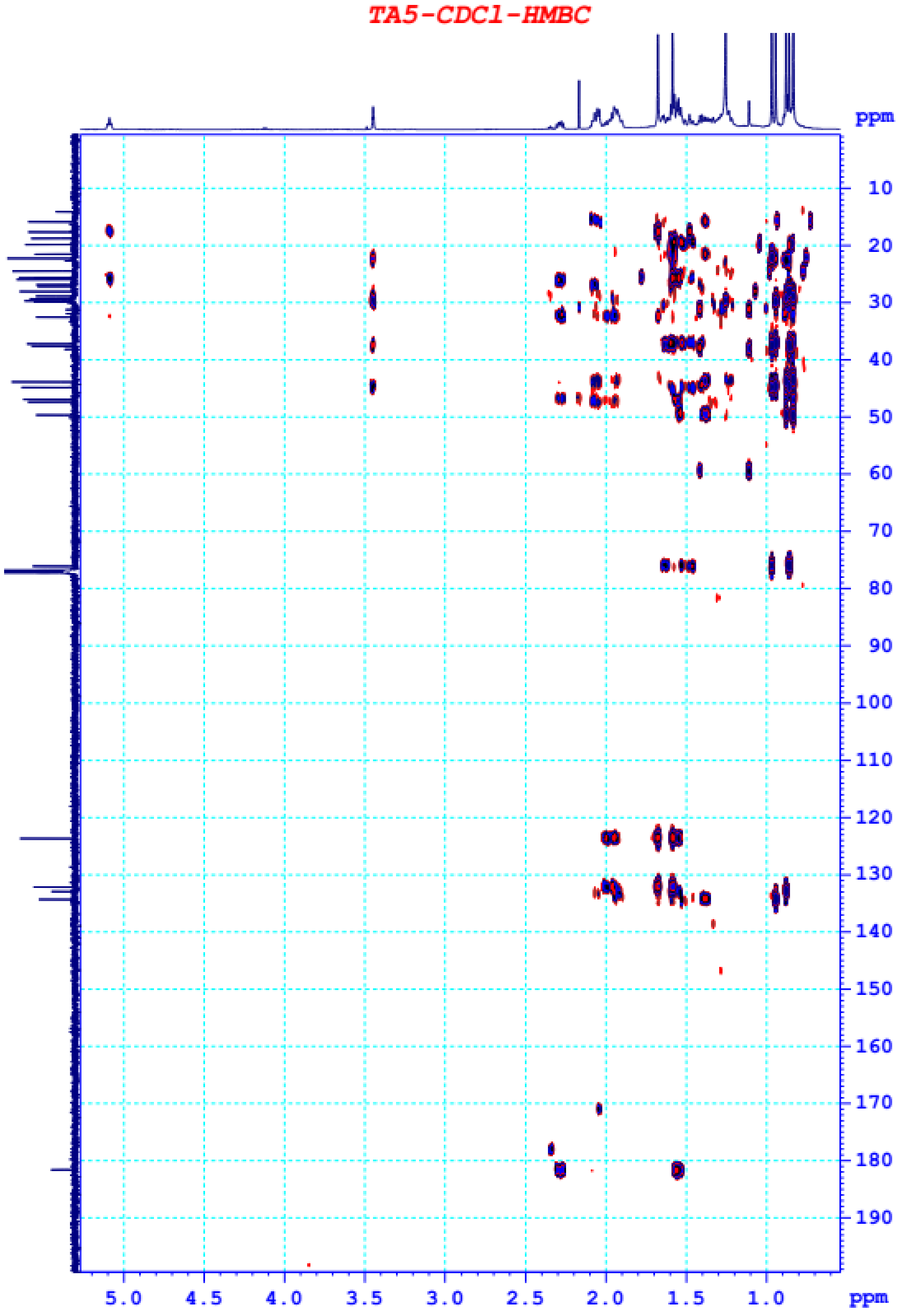

Figure S22. HMBC spectrum of compound **6**

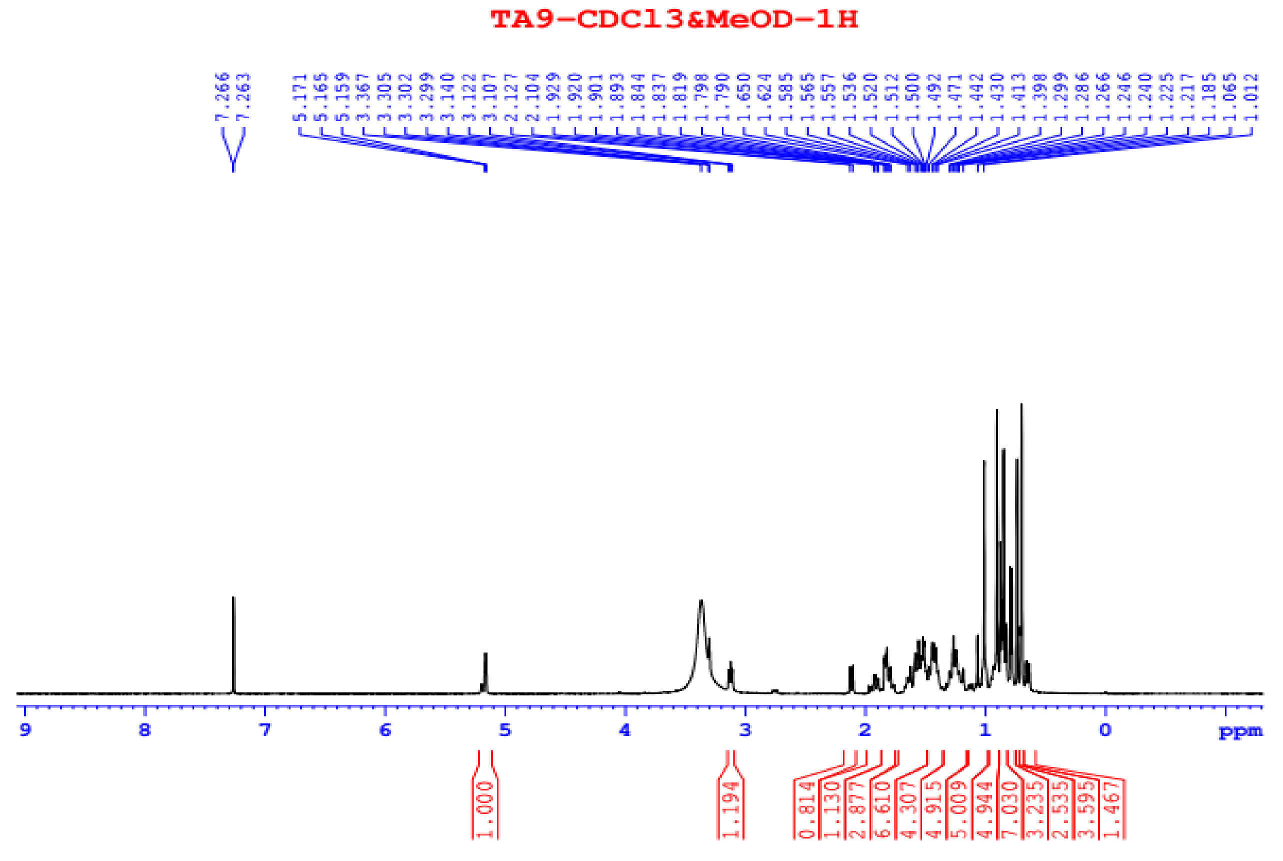

Figure S23. ^{1}H-NMR spectrum of compound **7**

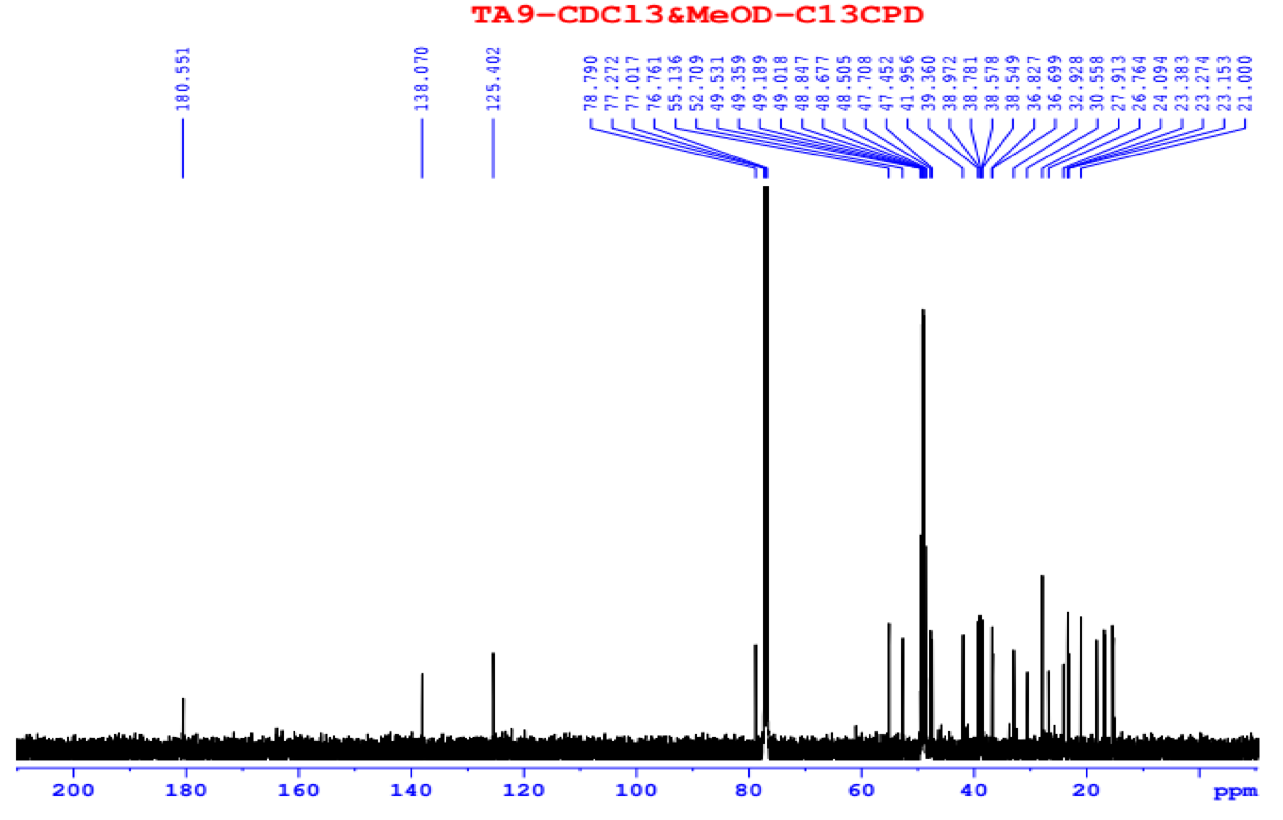

Figure S24. ^{13}C-NMR spectrum of compound **7**

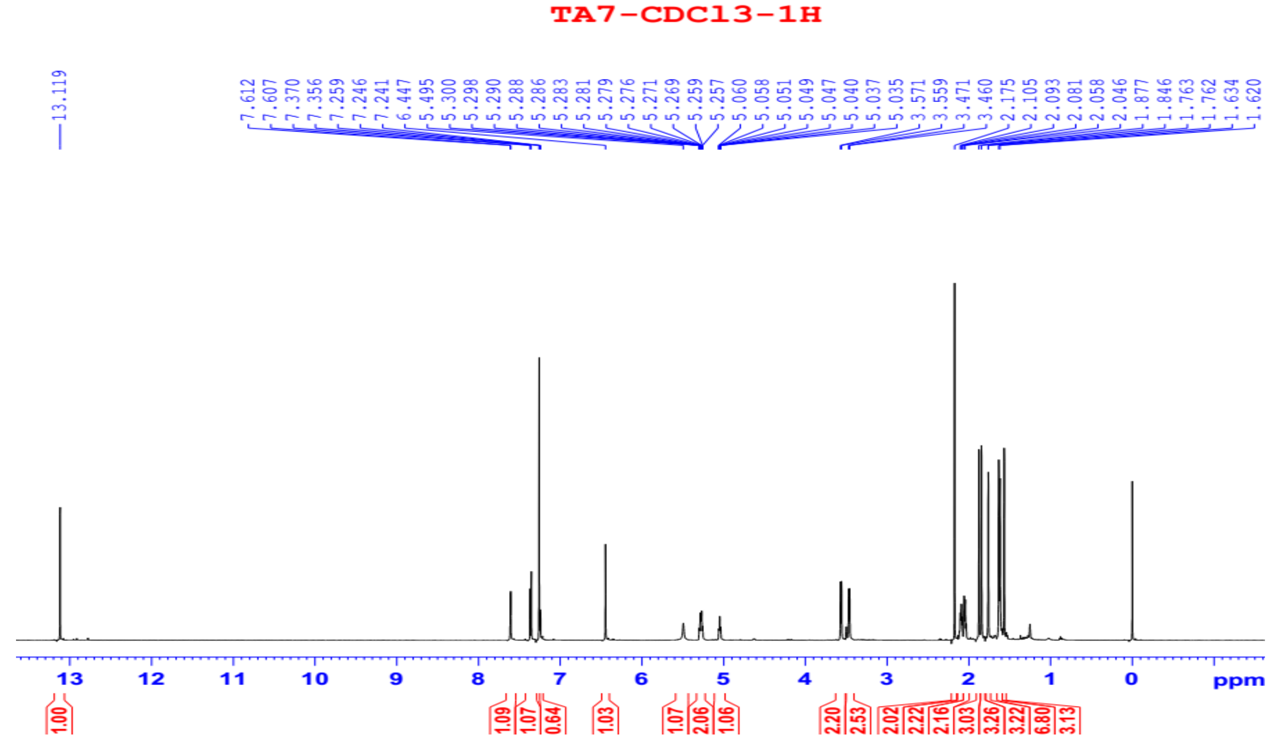

Figure S25. ^{1}H-NMR spectrum of compound **8**

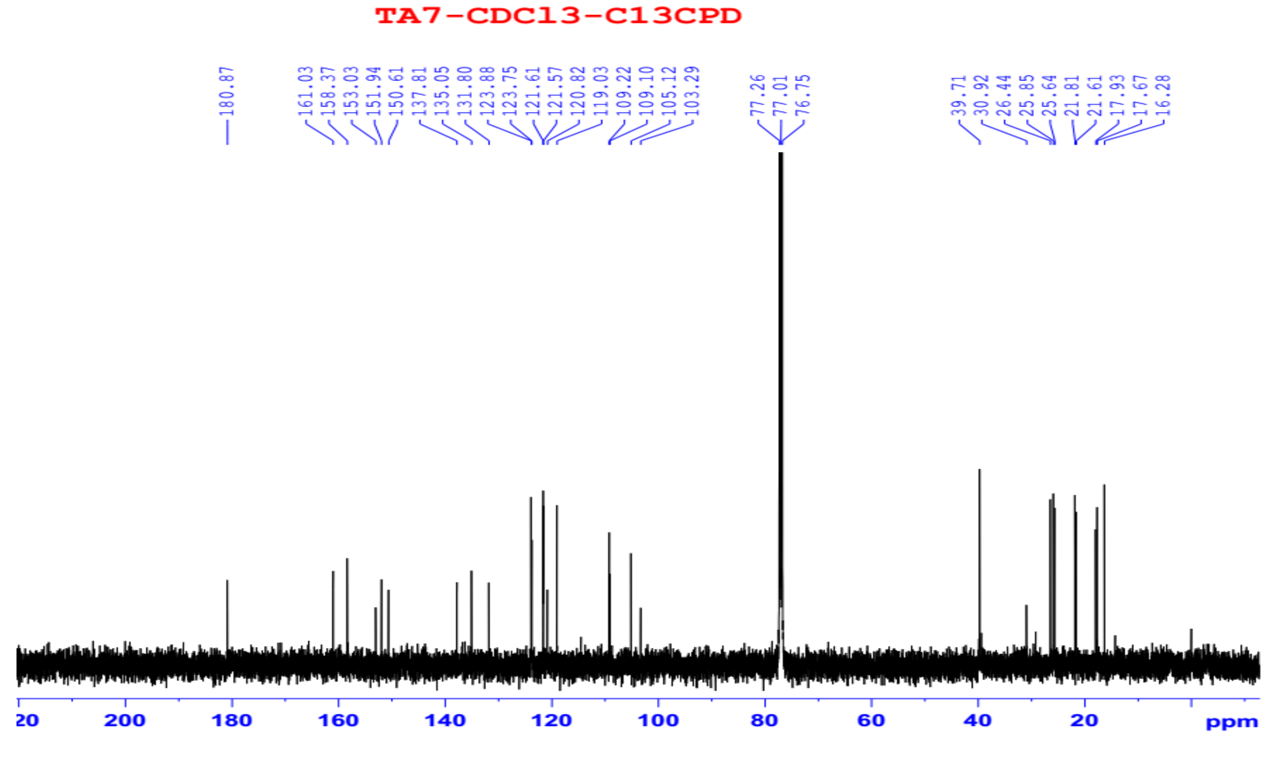

Figure S26. ^{13}C-NMR spectrum of compound **8**

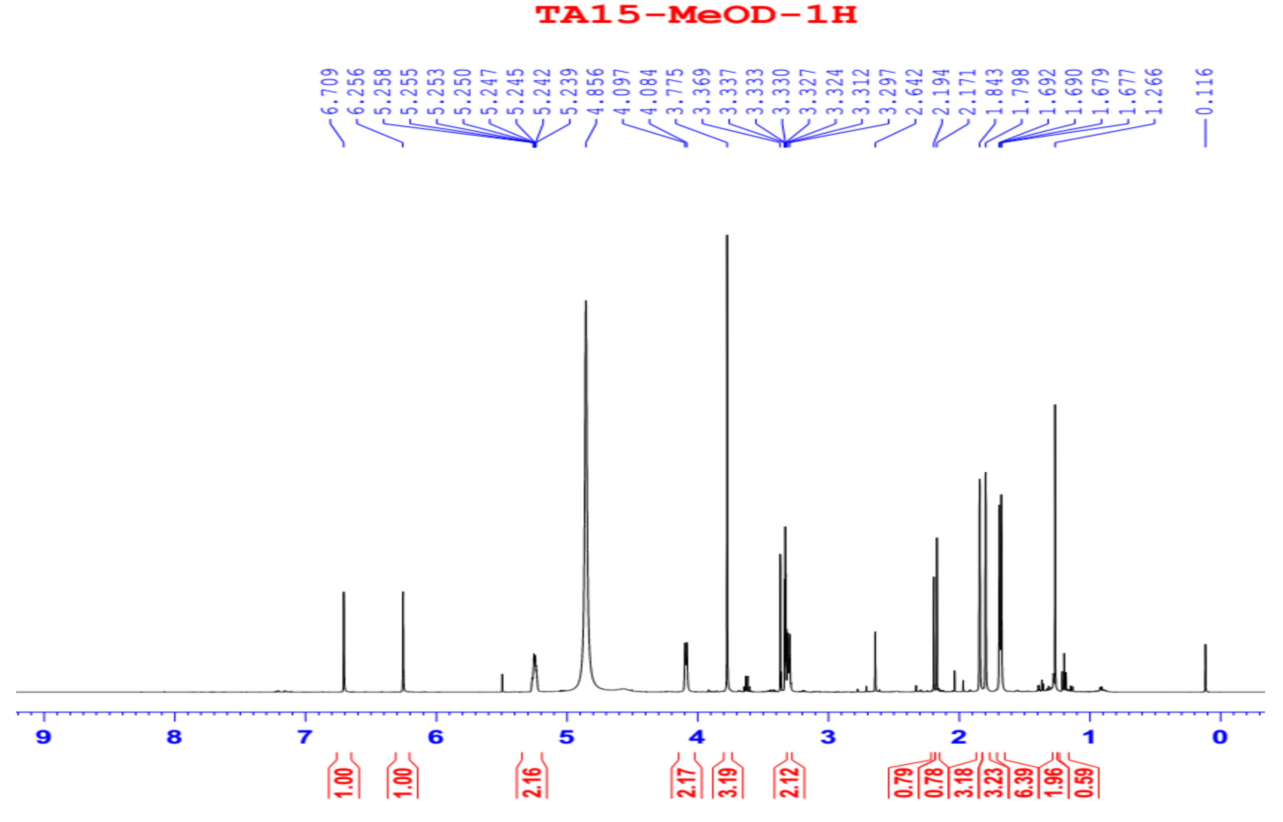

Figure S27. ^{1}H-NMR spectrum of compound **9**

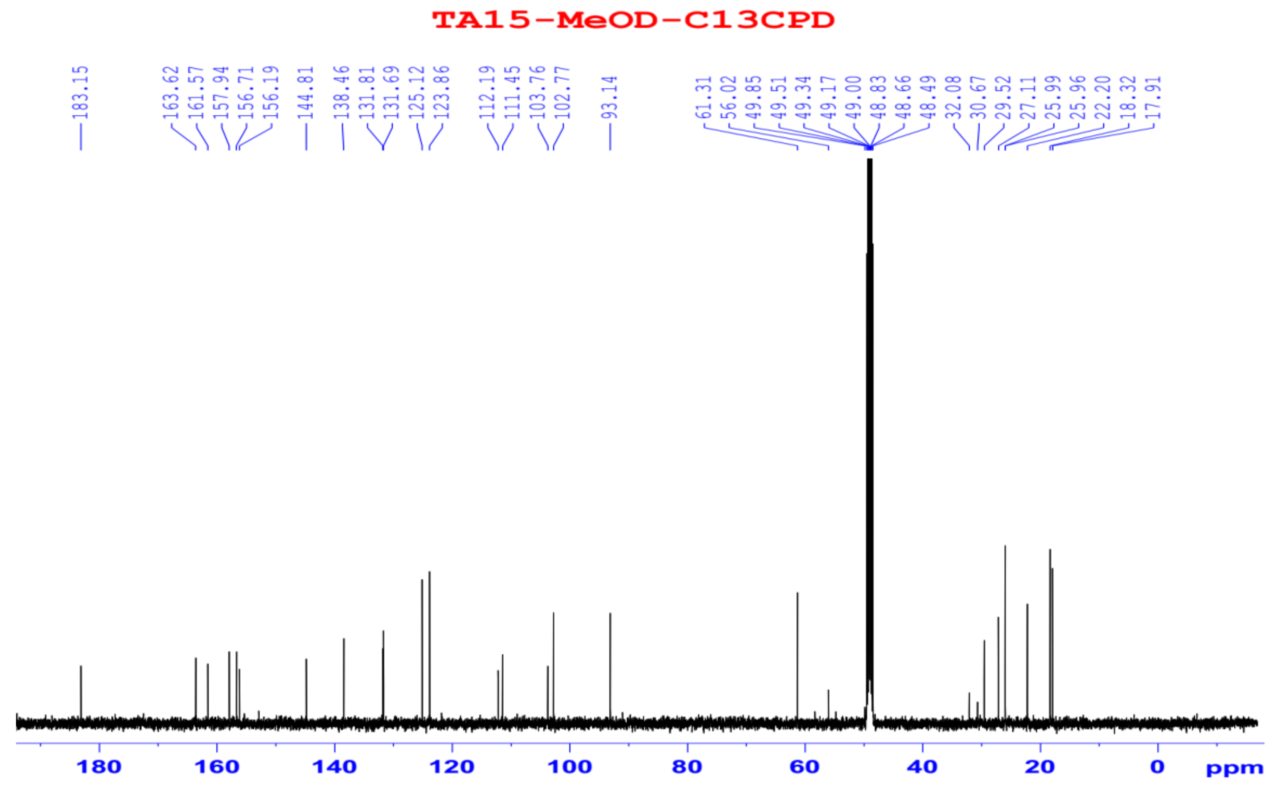

Figure S28. ^{13}C-NMR spectrum of compound **9**
